# Combining research and design: A mixed methods approach aimed at understanding and optimising inpatient medication storage systems

**DOI:** 10.1371/journal.pone.0260197

**Published:** 2021-12-02

**Authors:** Carly Wheeler, Alice Blencowe, Ann Jacklin, Bryony Dean Franklin

**Affiliations:** 1 Centre for Medication Safety and Service Quality, Imperial College Healthcare NHS Trust, London, United Kingdom; 2 NIHR Imperial Patient Safety Translational Research Centre, London, United Kingdom; 3 Helix Centre, Imperial College London and The Royal College of Art, London, United Kingdom; 4 UCL School of Pharmacy, London, United Kingdom; Flinders University School of Medicine: Flinders University Medicine, AUSTRALIA

## Abstract

**Background:**

Almost every patient admitted to hospital will receive medication during their stay. Medication errors are an important cause of patient morbidity and mortality, as well as an economic burden for healthcare institutions. Research suggests that current methods of storing medication on hospital wards are not fit for purpose, contributing to inefficiency and error.

**Aim:**

To improve medication storage in inpatient areas, by exploring variation and challenges related to medication storage and designing a prototype solution.

**Methods:**

Set in four hospitals in an English teaching hospital trust, the study used a mixed methods approach comprising a quantitative descriptive survey of storage facilities and practices followed by mixed methods observations of medication rounds and interviews with patients, nurses and pharmacy staff. Quantitative data were presented descriptively and qualitative data analysed thematically and using a human-centered design approach.

**Results:**

We identified wide variation in medication storage facilities and practices across 77 wards. Observations and staff interviews in six wards revealed five problem areas: poor management of multiple storage facilities; lack of visibility and organisation of medication within trolleys; inadequate size of storage; lack of ownership and knowledge of standard practice; and use of key locks. Patients were largely satisfied with receiving their medication. Systematic and consistent physical organisation of medication in medication trolleys, and integrating and implementing principles of best practice, were identified as areas for intervention.

**Discussion and conclusion:**

Variation in medication storage facilities and practices existed both across the organization and on individual wards. Multiple challenges were identified in how medication was stored, which if addressed may improve the efficiency and safety of medication administration and in turn, staff and patient experience. The use of design principles alongside a research approach resulted in a rapid, iterative process for developing and refining potential solutions to improve inpatient medication storage.

## Introduction

Medication is the most commonly used medical intervention, with nearly all hospital inpatients receiving medication during their stay. Unfortunately, medication errors do occur and may cause considerable patient harm, as well as being an economic burden for healthcare institutions [1–3]. Accordingly, in 2017 the World Health Organization (WHO) identified Medication Without Harm as the theme for its third Global Patient Safety Challenge [2]. This seeks to improve medication safety by strengthening systems for reducing medication errors and avoidable medication-related harm [2]. This approach highlights that medication administration does not occur in isolation, but occurs within wider systems, involving multiple stages, healthcare professionals, patients and carers. While there are risks throughout the entire medication process, errors occur most often during medication administration [2]. In a review of the English literature, 79% of secondary care errors were estimated to occur during dose administration [1].

Hospital medication storage and administration systems have changed considerably in the last twenty years. For example, historically in the United Kingdom (UK), medication was stored in a central medication room and/or a medication trolley that was used for transporting medication to each patient. Around 2000, bedside medication lockers became more common as an additional type of medication storage [4], with the aim of facilitating self-administration and the use of patients’ own drugs brought in from home. Considerable variation in storage facilities and practices across and within hospitals was subsequently described [5]. More recently, various technologies have been introduced to improve the safety and efficiency of medication administration, including the use of automated dispensing cabinets to store and manage medication.

Research examining medication administration errors has identified how the configuration of medication storage systems may contribute to efficiency, security, safety and patient experience [6] In an international systematic review of the causes of medication administration errors, issues relating to medication storage or supply were highlighted in 27 of 54 studies, including lack of ward stock leading to missed or delayed doses, as well as medication being lost or misplaced [3]. How medication is stored and subsequently prepared has also been identified as a barrier to patient engagement, with medication that is prepared away from the patient’s bedside reducing patient engagement with their medication [7]. Where medication was prepared at a patient’s bedside from a trolley, compared to from a drawer in a workstation immediately outside a patient’s room, Fischer et al. [8] also identified a lower medication administration error rate. Lack of an appropriate medication storage solution has also been described as a barrier to patients self-administering their own medication [9]. In summary, research therefore highlights how current medication storage does not meet the needs of healthcare professionals or patients.

An approach to generating solutions to problems that is increasingly used in health care is human-centered design [10]. Human-centered design is an approach to problem solving that considers the human perspective at all points, focusing on the users of a system, their needs and requirements [11].

Our overall aim was to improve medication storage in inpatient areas to facilitate safe and efficient medication administration. In order to achieve this, our objectives were: (1) to describe medication storage facilities and practices, and any associated variation among wards and hospitals, across a large UK teaching hospital organisation; (2) to explore the challenges associated with current medication storage and administration systems and identify areas for improvement; and (3) to design a prototype solution to address the challenges identified, using a human-centered design approach.

## Methods

A sequential explanatory mixed methods design was used, comprising a quantitative phase of data collection and analysis to address the first objective, followed by a predominantly qualitative phase of data collection with some embedded quantitative data to address the second and third objectives.

### Setting

The study took place at four of the five hospitals of a large teaching hospital trust in London, UK. The fifth hospital was excluded as it had few inpatient beds. The trust used an electronic prescribing and administration system for hospital inpatients; each nurse administered medications to their allocated patients. Systems for dispensing and administration of medication were typical of those in UK hospitals. Briefly, commonly used medication was kept as stock on each ward, with 1–2 week supplies of non-stock medication dispensed by the pharmacy department for individual patients as needed. If patients brought in supplies of their own medication this was used during their stay if it met criteria for suitability. Inpatients were permitted to administer their own medication if assessed as competent to do so. Medication storage for oral medication was not standardised, and barcode medication administration (BCMA) was not in use at the time of data collection. Nurses prepared medication at the time it was needed during each drug round.

### Phase one–descriptive survey (predominantly quantitative)

A descriptive, cross-sectional survey design was used. The survey (S1 Appendix) comprised 32 questions, both open and closed, exploring the types of medication storage facilities present on each ward, where different types of medication were stored, and which healthcare professionals were responsible for different aspects of medication storage. Only inpatient wards were included. Oral medication was the focus of the survey; intravenous medication, medication stored in fridges, and controlled drugs were excluded as these medications have separate requirements and policies regarding their storage. The surveys were completed either by a health services researcher or a pharmacist, with the assistance of the nurse in charge or his/her nominee, between August and November 2018. The health services researcher or pharmacist visited each ward, observing storage facilities and practices, with nursing staff providing clarification/additional information as needed.

#### Survey data analysis

Data were analysed descriptively, with data from both open and closed questions used to produce frequency counts and percentages using Excel.

This phase of the project was deemed to be a service evaluation and registered as such within the hospital trust (reference number 289); NHS ethics approval was not required.

### Phase two–observations (qualitative and quantitative) and interviews (qualitative)

The second phase of data collection comprised observations of medication administration rounds and interviews with nurses, pharmacy staff and patients relating to inpatient medication administration. Six wards were selected through purposive sampling based on the survey findings, aiming for maximum variation in clinical specialties, ward size, and medication storage systems. Observations and interviews took place between July and November 2019.

Focused ethnographic observations of medication administration rounds were conducted by a health services researcher, with three separate observations conducted with different nurses on each ward, on different days and at different times of day. Observations focused on the tasks, events and decision-making processes associated with medication administration and storage. Observation notes were recorded narratively during the observation and expanded upon afterwards, with reflections from the observer added as soon as possible after the observation. Some descriptive quantitative data were also collected on the number of patients receiving medication, the number of doses of medication given during the round, the number of doses administered by the nurse versus self-administered by the patient, omitted doses (not including doses declined by the patient), locations from which doses were retrieved, and the number of doses that were not found in the first place the nurse looked for them. ‘Spaghetti diagrams’ depicting nurses’ travel superimposed on the ward’s floorplan [6] were produced for each observation.

Additional observations, on two medication administration rounds, were conducted by a lay researcher (a member of the public) to capture other insights from a patient perspective. During these observations, the health services researcher who conducted the other observations was present on the ward but did not accompany the lay researcher on the medication round. The lay researcher was briefed as to the purpose of the study and asked to take unstructured notes on anything that stood out, was surprising or seemed of particular importance from her perspective. The lay researcher did not collect any quantitative data on numbers of doses or produce spaghetti diagrams of the medication round.

Individual semi-structured interviews were then conducted with key stakeholders involved in the storage of medication: nursing staff, pharmacy staff and patients. All healthcare professionals who were involved in medication administration on the study wards were eligible to be interviewed, as were patients (and/or their carers) over the age of 18 who were conversant in English and had received medication while an inpatient on the study wards. Interviews were guided by a topic guide (S2 Appendix), audio-recorded and transcribed verbatim.

Ethical approval for phase two was obtained from a NHS Research Ethics Committee (reference 19/LO/0863) and permission obtained from the nurse in charge for each participating ward. All participants provided written informed consent prior to observations and interviews.

#### Observation and interview data analysis

*Quantitative observational data*. Quantitative data from the observations were presented as means and standard deviations, or medians and interquartile range for non-parametric data.

**Fig 1:**
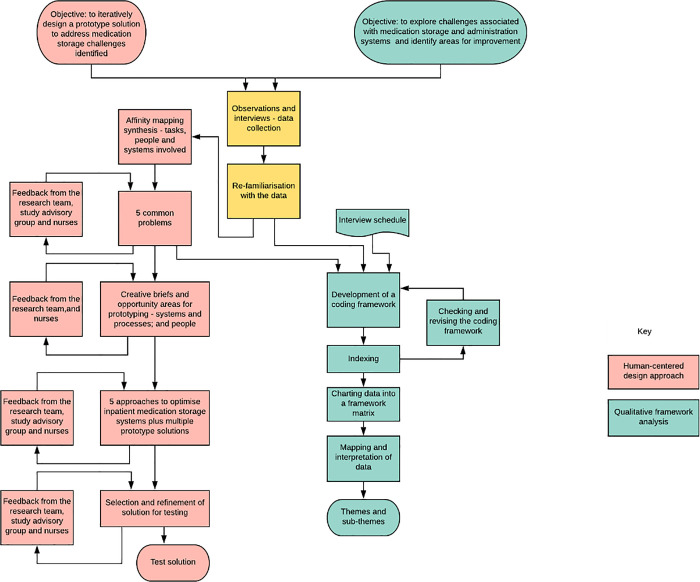
A flow chart depicting the analysis of observation and interview data using a both a human-centered approach and a thematic framework approach.

*Qualitative observational and interview data*. Qualitative data were analysed using both thematic framework analysis and a human-centered design approach (Fig 1).

*Thematic analysis*. Qualitative data from the observations and interviews were analysed thematically using a framework approach [12]. The first phase of the framework methodology was re-familiarization with the raw data and noting key ideas and recurrent themes. The data were then coded using both a deductive approach–guided by the interview topic–as well as inductively, allowing for the emergence of any new themes. The data were then rearranged and summarized into a framework matrix. CW coded the data and BDF independently reviewed a sample of observations and interviews plus the thematic framework, with any differences resolved through discussion.

*Human-centered design approach*. Qualitative data from both observations and interviews were synthesised according to the tasks, people and systems involved in the medication administration process, using affinity mapping. The quantitative observational data were also taken into consideration. This was done by CW and AB–a health services researcher and a designer—by re-reading and dissecting interview transcripts and observation notes. Major themes across the data were visually organised and coded, together with human insights, such as user needs or preferences, to produce a set of common problems relating to medication storage that spanned the end-to-end user journey from the point of view of nurses, patients and pharmacists.

The emerging set of common problems were discussed with the wider research team, a study advisory group (researchers, designers, pharmacists, nurses, quality improvement experts and members of the public) and nurses from participating wards. Through an iterative process, using the feedback gathered, the afore mentioned set of common problems relating to medication storage were used as the basis for developing two creative briefs with contrasting foci: one around storage systems and processes, and the other around people–their behaviours, motivations and ways of working. Within these creative briefs we identified opportunity areas for creating potential improvement solutions for testing. Following this iterative process, these opportunities were first discussed among the research team and ward nurses, and then alongside proposed solutions with the study advisory group. Advisory group members were asked to rank the proposed solutions in terms of importance, feasibility, likely cost, and perceived impact. Incorporating this feedback and following further discussion with nurses and the hospital’s quality improvement team, a prototype solution for testing was proposed and presented at the third and final study advisory group meeting.

### Patient and public involvement

The research project was presented to a”research partners’ group” prior to the start of the project, to identify appropriate involvement of patients, carers and members of the public at the authors’ institution. Suggestions for lay involvement in data collection and collaboration in analyzing/interpreting findings were subsequently incorporated through lay involvement in data collection as previously described, plus two lay members in our advisory group contributing to analysis and interpretation of the findings.

## Results

### Phase one–descriptive survey

All 77 inpatient wards were surveyed across four hospitals within the hospital trust.

#### Storage facilities

Storage facilities were viewed on each ward surveyed by either the health services researcher or pharmacist. Medication rooms were present in all four hospitals, in a total of 72 (94%) wards. Medication trolleys (including both standalone medication trolleys and computers on wheels with built-in storage) were present on 41 (53%) wards across all four hospitals. Bedside storage was present on 60 (78%) wards across the four hospitals surveyed. Other types of storage included automated dispensing cabinets on 2 (8%) wards. There was considerable variation among and within hospitals (Table 1).

**Table 1:** Medication storage facilities on wards across the four hospital sites.

Hospital site	Medication room	Trolley	Bedside locker	Other(s)
1 (n = 24 wards)	24 (100%)	9 (38%)	24 (100%)	2 (8%)
2 (n = 17 wards)	16 (94%)	4 (24%)	16 (94%)	2 (12%)
3 (n = 28 wards)	24 (86%)	19 (68%)	19 (68%)	5 (18%)
4 (n = 8 wards)	8 (100%)	7 (87.5%)	1 (12.5%)	1 (12.5%)
Total (n = 77 wards)	72 (94%)	41 (53%)	60 (78%)	10 (13%)

#### Storage practices

Data on storage practices was collected through a combination of the health services researcher/pharmacist witnessing practices on each ward when visiting to complete the survey, and self-report by the nurse in charge or their deputy. Ward stock medication was most frequently reported as being stored solely in a medication room (35 of 77 wards; 45%) or in both medication rooms and trolleys (35 wards; 45%). Medication dispensed to individual patients for inpatient use was most frequently reported as being stored at the bedside (57 wards; 74%). Patients’ own medication was also most frequently reported as being stored at the patient bedside (56 wards; 73%). Again, there was variation among and within hospitals (Table 2).

**Table 2:** Primary storage locations for ward stock, individually dispensed medication and patients’ own medication on all wards across the four hospital sites.

	General ward stock				Individually dispensed medication				Patients’ own medication			
	Site 1 (n = 24 wards)	Site 2 (n = 17 wards)	Site 3 (n = 28 wards)	Site 4 (n = 8 wards)	Site 1 (n = 24 wards)	Site 2 (n = 17 wards)	Site 3 (n = 28 wards)	Site 4 (n = 8 wards)	Site 1 (n = 22 of 23 wards)^a^	Site 2 (n = 17 wards)	Site 3 (n = 25 of 28 wards wards)^b^	Site 4 (n = 6 of 8 wards)^c^
Medication room	15 (63%)	13 (76%)	7 (25%)	0	0	2 (12%)	6 (21%)	2 (25%)	0	1 (6%)	4 (16%)	1 (16.7%)
Trolley	0	0	0	0	0	0	2 (7%)	3 (37.5%)	0	0	0	0
Bedside locker	0	0	0	0	23 (96%)	15 (88%)	18 (64%)	1 (12.5%)	22 (100%)	16 (94%)	16 (64%)	1 (16.7%)
Combination of locations/ other	9 (38%)	4 (24%)	21 (75%)	8 (100%)	1 (4%)	0	2 (7%)	2 (25%)	0	0	5 (20%)	4 (66.7%)

^a^ one ward reported not storing patients’ own medication on the ward.

^b^ three wards reported not storing patients’ own medication on the ward.

^c^ two wards reported not storing patients’ own medication on the ward.

When medication was transported from a ward storage area to a patient’s bedside during the drug round, it was most frequently reported by the nurse in charge as being transported by a trolley (32 wards;42%) or on a handheld tray (26 wards;34%). Other methods of transport reported included ‘by hand’ or using a combination of methods. Medication was prepared solely at the bedside on about half of the wards (42 wards; 55%).

### Phase two–observations, interviews, and prototype solution development

Details of the six participating wards are presented in Table 3. Eighteen medication rounds were observed by a health services researcher, with durations ranging from 10–120 minutes (Table 4). The lay researcher observed an additional two rounds. The mean number of patients receiving medication on the observed rounds was 3 (standard deviation 1.8; range 1–7). The median number of doses of medication administered was 8 per round (interquartile range: 12; range 1–48). Five patients on ward A self-administered their own medication.

**Table 3:** The six wards on which observations were conducted and from which staff and patients were recruited for interview.

Ward	Hospital site	Specialty	Number of beds	Types of medication storage
A	1	Neurology	5	Medication room; bedside lockers
B	1	Oncology	26	Medication room with an automated dispensing cabinet; bedside lockers
C	2	Haematology	14 (all individual rooms)	Medication room
D	2	Renal	16	Medication room; trolleys; bedside lockers
E	3	Orthopaedic surgery	30	Medication room; trolleys; bedside lockers
F	3	Bariatric and general surgery	14	Medication room; trolleys; bedside lockers

**Table 4:** Descriptive data from six wards collected during medication round observations.

Ward	Medication round duration (min)	Patients who received medication	Doses administered by nurse (Doses self-administered by patient)	Mean ± SD or median (IQR) doses per patient	Omitted doses	Doses from medication room	Doses from trolley	Doses from a bedside locker	Doses from other location	Doses not in the first location looked in
A	20	3	21 (1)	7.3 ± 3.5	0	1	No trolley	(21)^d^	0	0
A	10	1	4	4.0 ± 0.0	0	2	No trolley	2 (1)^d^	0	2
A	10	2	10 (1)	5.5 ± 2.1	0	1	No trolley	10	0	0
B	20	2	4	2.0 ± 1.4	0	3	No trolley	1	0	3
B	10	1	1	1.0 ± 0.0	1	0	No trolley	1	0	0
B	120	4	26	5.0 (4.0)	3	15 (1)^b^	No trolley	11	0	6
C	10	1	1	1.0 ± 0.0	0	1	No trolley	No locker	0	0
C	35	3	26	8.7 ± 4.2	1	26	No trolley	No locker	0	0
C	35	4	8	2.0 ± 0.8	2	7	No trolley	No locker (1)^d^	0	0
D	90	7	49	7.0 ± 2.0	2	6(1)^b^	24	15	4 (3)^e^	4
D	85	3	6	1.0 (-)^a^	1	1	3	2	0	1
D	10	2	6	3.0 ± 1.0	1	2 (1)^b^	3	1	0	1
E	40	5	14	2.8 ± 0.8	1	7 (5)^b^	6 (1)^c^	1 (1)^d^	0	1
E	30	5	9	1.0 (2.0)	3	0	9 (1)^c^	0	0	1
E	25	2	8	4.0 ± 1.4	1	1	5(3)^c^	1	1	3
F	40	3	6	1.0(-)^a^	3	0	5 (1)^c^	1	0	2
F	50	6	13	2.2 (1.9)	2	7(7)^b^	2	4	0	0
F	35	5	7	1.0(1.0)	2	0	6	1	0	0

SD = standard deviation; IQR = interquartile range.

(-)^a^ indicates that the median was based on three patients and so the IQR could not be calculated.

(x)^b^ indicates the number of doses that were retrieved from the medication room at the start of the round, either in preparation for the patient or for another patient, but at the time of administering were in a trolley or tray.

(x)^c^ indicates the number of doses retrieved from another trolley.

(x)^d^ at the bedside but not in a bedside locker.

(x)^e^ indicates the number of doses that were retrieved from another location at the start of the round, either in preparation for the patient or for another patient, but at the time of administering were in a trolley or tray.

Interviews lasted on average 20 minutes (range 7 to 42). Ten nurses were interviewed: two each from wards A, B, C, E and F. Of these, three were senior nurses and seven were staff nurses. Three members of pharmacy staff were interviewed: a senior pharmacist and a pharmacy technician from ward B, and a junior pharmacist from ward D. Twelve patients were interviewed, two from each of the six wards. Seven were female and five male, with a mean age of 57 years (range 40–77). All were inpatients, one of whom had an arrangement for temporary discharge and went home most nights, returning the following morning. Seven were receiving medication for long-term conditions and were in hospital for that condition. A further two patients were receiving medications for long-term conditions but were in hospital for a different reason. Three of the twelve were self-administering oral medication during their current hospital stay. No carers were interviewed.

#### Challenges associated with medication storage

The first step of the human-centered design approach resulted in identification of five broad problem areas. The structured thematic analysis identified the same challenges with medication storage. These are therefore presented together, combining findings from both approaches to analysis.

##### 1. Navigating a system of multiple storage facilities and practices

Only one of the six wards reported storing medication in only one location (Table 3), two were storing medication in two locations and three in three locations. Both within a single ward and across wards there was variation in where different types of medication were located. For wards with multiple storage facilities, stock medication was spread across these, with commonly used medication often in trolleys and stock in use by a particular patient sometimes kept at their bedside. On wards with bedside storage, patients’ own medication from home was generally kept at the bedside.

Ten of the 18 observed medication rounds revealed nurses not finding at least one dose of medication in the first place they looked for it (Table 4); nurses sometimes had to search in multiple locations, such as the patient’s bedside locker, the medication room, their own trolley and/or other trolleys. Such searching in multiple locations resulted in increased travel around the ward with frequent interruptions from staff, patients and relatives, as well as the nurse searching for medication interrupting other nurses.

“*I think when you’re a regular member of staff for a long time here*, *you do start remembering where things are kept and where things should be*. *But I can understand for new members of staff and bank and agency nurses*, *I can see how it can get confusing where things would be…everything’s quite dotted around the ward so it can get quite confusing for new people*.*” (Nurse 2*, *Ward E)*

**Fig 2:**
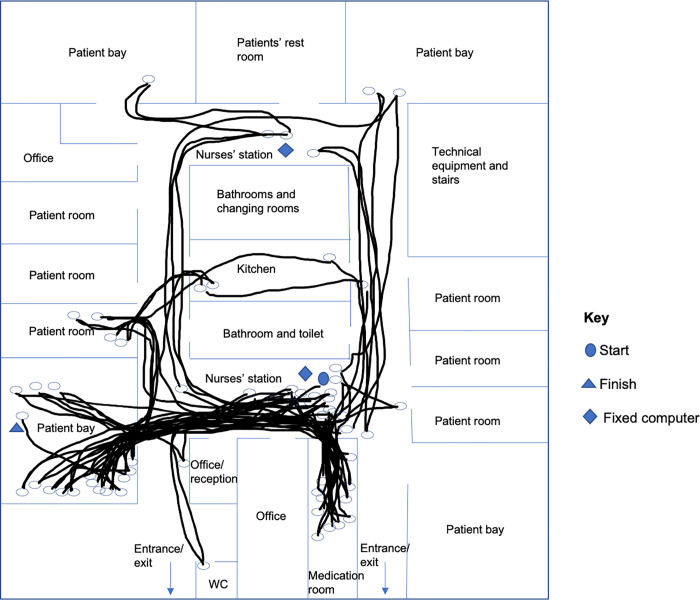
A spaghetti diagram showing nurse travel around ward B. Medication round duration: 120 minutes; number of patients who received medication: 4; number of doses administered: 26.

To support infection control on the haematology ward (ward C), medication was stored only in the medication room. This was the only ward on which all doses were located in the first place in which the nurse looked for them. A drug round of 26 doses of medication on ward C took 35 minutes, whereas a drug round also of 26 doses on ward B took 120 minutes, including six occasions where the nurse did not find medication in the first place they looked for it (Table 4). Fig 2 shows the travel of the nurse on ward B during this round.

Wards also varied in how trolleys were used. While the general consensus among nurses was that medication should be largely the same across multiple trolleys on a ward, medication was often missing from one trolley and needed to be obtained from another. On ward F, medication dispensed to individual patients and patients’ own medication were also stored in the trolleys in cases where patients’ bedside lockers were not operational due to broken locks. On this ward, one trolley was allocated to each half of the ward, however there was no labeling of the trolleys to identify which was which. Nurses were observed starting a medication round with the ‘wrong’ trolley for their patients and later needing to retrieve their patients’ medication from the other trolley.

On wards D and E, trolleys were taken around the ward on a ‘first come, first served’ basis, as nurses tried to get a “good one,” namely a trolley believed to be the best stocked. Nurses also described trying to get the same trolley used on a previous shift as they then knew what medication was inside.

##### 2. Organising medication within medication trolleys

On all three wards using medication trolleys to store and transport medication, challenges were both observed and described in relation to how to organize them.

Three different types of medication trolley were in use (S3 Appendix) in varying numbers across four wards.

Within the trolleys, medication was organised differently both within and across wards. The two nurses observed on ward F used the trolleys differently, with one nurse using the trolley as she found it and a second altering its organisation to meet her needs:

*“Everyone’s different*. *I’m dyslexic so first thing when I come in*, *I make sure I put everything the way I like so once I leave*, *they might be back to whatever else*. *So some people do antibiotics one side*, *analgesia one side*, *antiemetics one side but*, *for me*, *I’ll just put whatever works for me*. *So it might be alphabetical order*, *might be boxes level or colours*, *so yeah*. *So when I start the shift*, *before I start my drug rounds*, *I organise my trolley and I keep the keys*, *just this one*, *keep the keys until I finish my shift so nobody messes with it*.*”* (Nurse 2, Ward F).

On wards D and E, the two-drawer computer on wheels trolleys had little formal organisation. The smaller top drawer was generally used to store the most frequently used medication such as paracetamol and loose strips of other medication, with the larger bottom drawer holding bigger boxes, sometimes bottles of liquids and other medications. Some nurses indicated that medication should ideally be in alphabetical order within the trolleys, but that it rarely stayed that way.

Lack of organisation in the trolleys often meant nurses spent time searching for medication within the trolley, involving searching in multiple drawers and/or for medication that was not clearly visible. In one example on ward E, when a nurse realized she did not have the medication she needed in her trolley, she proceeded to look in three other trolleys, some of which were in use by other nurses, the medication room and then back to the first of the other nurses’ trolleys she had looked in, where she then located the medication.

On ward E, the newer six-drawer trolleys had labeled drawers corresponding to different groups of medication e.g. analgesia, antibiotics. Some drawers were labeled with two different categories of medication with no divide between them. These trolleys were universally preferred:

*“The trolleys with different drawers*, *you can organize by the analgesia*, *so you can put in order*. *But the normal trolley is more difficult because you just have one drawer so all the medication is altogether and all mixed”*. (Nurse 1, Ward E)

*“I just find them so much easier to use*, *they’re just more organised*, *there’s not as much stuff in them as there is in the other ones*.*”* (Nurse 2, Ward E).

##### 3. Amount of medication storage

Not having enough space to store medication was a theme running across all wards. However, this was particularly apparent for bedside medication lockers. Both observed and reported by nursing and pharmacy staff, this had implications in terms of waste and medication being left past its expiry date, liquid medication leaking due to bottles being stored on their side, patient safety if medication had to be left unlocked at the bedside, and/or in terms of patients self-administration, if medication that would not fit in the bedside medication locker had to be stored away from the bedside:

*“If the patient is on a lot of drugs in the bedside locker it won’t all fit and you can’t put big bottles in there*. *So if they’re taking liquid medication it has to be kept out by the bedside which isn’t the safest thing*. *And then sometimes it gets put in the medication room where there isn’t a dedicated space for that*.*”* (Pharmacist, ward B)

Moving medication from the bedside locker to a general space in a medication room or trolley was seen to run the risk of medication getting mixed up, and adding additional clutter to often crowded medication rooms, where old medication may not be disposed of or patients’ medication could not be found.

As well as being difficult to store in bedside lockers, liquids and large items were also challenging to store in trolleys. Some medium-height containers would fit in the bottom drawer of the two-drawer trolleys, but not in the traditional computerless ones. In these instances, liquids had to be kept in the medication room.

Not having enough trolleys was also a challenge, particularly on wards using computers on wheels. Not only did nurses have to ‘compete’ with other nurses for trolleys to carry out their medication round, but also with doctors who needed a computer. Nurses described either having to wait for a trolley to perform their medication round or occasionally breaking with protocol and performing the medication round without one, which made preparation at the patient’s bedside challenging and introduced more travel around the ward.

While wards had varying sizes of medication room, the majority of nursing staff interviewed felt these did not have enough space to store medication and/or that the room itself was not large enough to allow preparation of medication by more than one nurse at the same time. Ward B was the only ward using an automated dispensing cabinet (Table 3), with its finite capacity limiting the amount of stock that could be stored as well as having only one cabinet resulting in queuing to access medication.

##### 4. Key-lock medication storage

All six wards were using keyless access to their medication rooms: four with digital locks and two with swipe-card access. However, some medication trolleys and bedside lockers had key locks, which were unanimously described as challenging, except for the newer trolley variation on ward E, with the latter having a digital lock. Nurses were observed travelling back and forth between different areas of the ward and interrupting other staff to retrieve keys to trolleys, to unlock and lock trolleys at the start and end of medication rounds, and also during the round if they needed to leave the trolley unattended. On some observations nurses left trolleys unlocked and unattended, instead of locating the key. On ward F, which had two trolleys, nurses were observed and also reported picking up the wrong key for the trolley they wanted and not realizing until they came to try and unlock it.

*“All of our trolleys are done by keys*, *so trying to find the nurse with the keys can sometimes be a bit of a pain*. *Just to unlock them or lock them and things like that*. *But that’s just how it’s done and you do eventually find them*, *but it can take a little bit longer sometimes*.*”* (Nurse 2, Ward E)

Four of the five wards with bedside lockers had digital, keyless locks. Ward D was using a combination of key and digital locks. Not only did key locks present a problem for nurses during the medication round, but additionally for pharmacists when checking medication or discussing it with a patient, as they then needed to find and interrupt the nurse who held the keys.

Keys were also observed and described as getting bent and therefore not working, or going missing–falling out of pockets or being taken off the ward.

*“Sometimes it can be difficult*, *well it’s more difficult for me with patient lockers that have a key*. *Because then I have to get hold of a key from a nurse and then obviously getting that back to them in a timely manner…and recently I was on the ward and I borrowed a key to check what was in the locker and then I took it home*. *It’s an hour and a half commute and I was half an hour from home and I realized I had the key in in my shirt pocket”*. (Pharmacist, Ward D)

##### 5. Alignment, knowledge and ownership of responsibilities and practice

Across wards B, D, E and F, a lack of clarity of, and/or lack of adherence to guidance was observed. Restocking and organisation of stock medication was often reported to be the role of nursing staff working night shifts. However, this was not always possible due to workload. Conversely, on the same wards, other nurses also reported not knowing who was responsible for organising stock. Lack of clarity was also true for pharmacists:

*Sometimes when a patient goes home and then there’s any medication that’s left in the locker*, *that can sometimes be left behind*. *So when the new patient arrives in the bed*, *there’s medication that shouldn’t be there in their locker”* (Pharmacist ward D)

Interviewer: *“And who would be responsible for removing that*?*”*

*“I’m not sure but*, *yeah*. *I don’t know what the process is actually*.*”* (Pharmacist ward D)

Nurses described how it was every nurse’s responsibility to top up medication as they go along if something runs out, such as during a medication round. However, in numerous instances nurses were observed arriving at a bedside locker or opening a trolley to discover that a medication was not there. A tension between nurses acting individually, versus for the collective team, was also described by interviewees:

*“The nurses [are responsible for keeping the computers on wheels organized]*, *we try*. *We got some nurses that are better than others at tidying up*. *If there’s empty boxes we are expected to throw them away*. *Not everybody does which is frustrating*.*”* (Nurse 2, ward E)

Another described how their effort to tidy and reorganise the trolleys on their ward had little sustained impact:

*“I have removed all the medications from the trolley and tried to organise all the medications*. *But the following day it was completely chaos again*.*”* (Nurse 1, Ward E).

Conversely there were a small number of instances where other staff involvement had been a hindrance. For example, during an observation on ward D, when asked by a student why there was medication missing from the medication trolley, resulting in multiple trips to the medication room to collect medication, the nurse inferred that medication had been removed during “cleaning” by night staff.

Other reported examples of staff uncertainty included doubts about whether patients’ own medications could be stored in the medication trolley, and for non-permanent members of staff e.g. bank staff, a lack of clarity as to where different types of medication should be stored. Even for permanent members of staff, knowing whether a medication was a stock medication or not was not always straightforward, with this having implications for staff not knowing where to locate such medications and how to replenish them:

*“Sometimes I’m not very sure if it’s stock or not*, *whether we should have that*. *And sometimes I might write in the pharmacy book that we need it and then people will say it’s stock*, *and so it won’t get ordered if it’s stock*.*”* (Nurse 1, Ward F).

The two pharmacists interviewed both spoke about the benefits of having a medication management technician on the ward who was clearly responsible for maintaining medication supplies. Nursing staff across all wards however were less clear on pharmacy staff involvement beyond the pharmacist and the roles of others who came to the ward.

#### Patient desires and experiences

Patients generally described being satisfied with their experiences of receiving medication during their stay. Among patients there were various degrees of knowledge regarding where their medication was stored. Some described a range of locations including bedside lockers, trolleys, a medication room, or a combination of these; others did not know where medication was stored if it was not at their bedside. Linked to this, patients described a mix of medication being prepared at their bedside and being brought to them already prepared as tablets in a small paper cup.

When asked, patients stated that on the whole they were not overly concerned as to where their medication was stored. However a number of relevant themes emerged that were of importance to patients: patients’ ability to self-administer medication, a desire for information, and receiving medication on time when it is due. Further detail on each of these themes is presented in S4 Appendix.

#### A prototype solution to address medication storage challenges

Following on from the five challenges related to medication storage, as described above, five potential opportunities to optimise inpatient medication storage were generated (Table 5).

**Table 5:** Opportunity titles and descriptions for a prototype solution for testing to address the medication storage challenges found.

Opportunity title	Opportunity description
‘Tidy trolley, tidy mind’	Promoting systematic and consistent physical organization of medication in storage facilities
‘Fail to prepare, prepare to fail’	Quick and easy preparation of a medication round
‘Don’t be kept in the dark’	Up to date information on medication stock levels and location on the ward
‘It’s all about people’	Effectively communicating and facilitating adherence of roles and responsibilities
‘Practice makes perfect’	Integrating and implementing effective best practice principles

Two opportunity areas were subsequently selected for further exploration, with a focus on having systematic and consistent physical organization of storage facilities along with knowledge, alignment and ownership over best practices, through the development of a two-part prototype solution (S5 Appendix).

Part A consisted of a set of standardised best practice principles for storing medication, creating a common language for ward managers, nurses and pharmacists regarding the practice of medication storage.

Part B was a data-driven and personalized five-step interactive guide to help wards set up their medication trolleys. By empowering ward staff with pre-prescribed options for how medication in their medication trolleys could be categorised (e.g. alphabetical, frequency of use), this could show users of the system ‘what good could look like’. Furthermore, by giving users the tools to collaboratively build their own system of medication organisation and apply it to their trolleys, using personalised laminated sticker sheets supported by a well-designed trolley ‘map’ and inventory, it was envisaged that this would improve medication processes in a number of ways. These include nurses being able to find medication quicker due to the improvement in organization and visibility of medication, making medication rounds more efficient through time savings, and reducing opportunities for interruptions when searching for medication. This would also contribute to meeting patients’ needs of receiving medication on time.

## Discussion

This study uniquely combined mixed methods research and design approaches, to explore how medication is stored on inpatient wards and involving the stakeholders in medication administration, to suggest methods of improvement. Across a single NHS hospital trust, considerable variation was evident in the facilities available for storing medication and how these were used.

A number of problems or challenges relating to medication storage were identified. These challenges most widely related to the poor system management of multiple storage facilities and practices, as well as a lack of organization of medication within a single piece of storage, namely medication trolleys. This lack of organization and subsequently visibility, of medication within medication trolleys echoes previous findings [6] that medication retrieval during the medication rounds was facilitated on wards where the front of medication packs were visible to aid identification. Instances of nurses travelling around the ward to obtain medication from multiple locations have also been reported [6]. In the present study, on five of the six wards, nurses were observed not finding medication in the first place they looked for it, sometimes resulting in travel away from the patient bedside to retrieve medication from elsewhere.

Patients described being satisfied with receiving their medication, however two key aspects linked to this satisfaction could potentially be influenced by medication storage. Firstly, patients’ desire for information and to be active in checking their medication could be limited when medication was not prepared at the bedside. Secondly, timely administration of medication could be enhanced if nurses were able to find medication when they need it.

### Implications for practice

This study identified a number of challenges associated with medication storage. It also suggests the importance of consulting those involved with medication administration when making changes to practice. The next step will be to implement and test the initial prototype solution developed through this work. As there is a shift to greater use of technology such as BCMA in medication administration, it will also be important to evaluate the impact of such changes. Additionally, the importance of offering patients the opportunity to be involved in their medication, whether through self-administration or checking medication prepared for them, may lead to improved patient satisfaction.

### Strengths and limitations

A strength of this research project was the large number of wards surveyed during phase one, which was used to inform phase two recruitment in which we selected wards with as wide a variation as possible. The interdisciplinary approach to this work and collaboration between research and design disciplines allowed for a more effective and holistic approach to meet the study aim and objectives.

A limitation is that we studied only one hospital trust and so the generalisability of our findings to other hospitals is unknown. However, previous work has also identified considerable variation across hospitals trusts in the English NHS, with medication trolleys associated with the most intra-hospital variation (McLeod et al., 2014). This suggests that our findings are not atypical. A further limitation is that it was not possible to develop one solution that addressed all of the challenges identified, for example further work will be needed to optimise bedside storage to facilitate inpatient self-administration. This reflects the complexity of the healthcare environment and a potential challenge and consideration for future collaboration between design and healthcare disciplines.

## Conclusion

We identified considerable variation in medication storage facilities and practices, with multiple challenges identified by nursing and pharmacy staff. Taking steps to improve these problem areas may improve the efficiency and safety of medication administration and in turn, result in better patient experience. Potential strategies for improving medication storage and subsequently medication administration include increasing ownership over storage facilities and systematic, consistent and visible storage of medication across medication facilities. We found that the use of design input alongside a research approach resulted in a rapid, iterative process for developing and refining potential solutions to improve inpatient medication storage, however the effectiveness of these solutions still needs to be tested.

## Supporting information

S1 Appendix Phase one survey. (DOCX)

S2 Appendix Phase two staff and patient interview topic guides. (DOCX)

S3 Appendix Photographs of medication trolleys. (DOCX)

S4 Appendix Patient desires and experiences. (DOCX)

S5 Appendix Prototype solution. (DOCX)

S6 Appendix Anonymised survey data from phase one. (DOCX)
