# Supplementary material for: Combining research and design: A mixed methods approach aimed at understanding and optimising inpatient medication storage systems
Source: PLoS One. 2021 Dec 2;16(12):e0260197. doi: 10.1371/journal.pone.0260197 (PMC8638963; doi:10.1371/journal.pone.0260197)
Supplement: S1 Appendix — (DOCX) [file pone.0260197.s001.docx]

**S1 Appendix 1 – phase one survey**

**MEDICATION STORAGE – SERVICE EVALUATION – DATA FORM**

**Hospital site:** ……………………………………. **Ward:** …………………………………….

**Specialty** ………………………………… **Number of beds:** …………………………………….

**Completed by:** ……………………………………. **Date:** …………………………………….

| **Question** | **Answer**  (please complete/delete as applicable) |
| --- | --- |
| **Storage facilities** |  |
| **Q1. Medication/treatment room** |  |
| 1(a) Is there a medication/treatment room? | Yes/no |
| 1(b) Whereabouts on the ward is the room located in relation to the nurses’ station? |  |
| 1(c) What is the approximate size of the room? |  |
| 1(d) Room lock type | None/digital lock/key lock/swipe card/RFID/other………………..(please specify) |
| 1(e) Are the cupboards within the room lockable? | Yes/no/some/no cupboards |
| 1(f) Who is responsible for organising medication in the room? |  |
| 1(g) Who is responsible for unpacking ward boxes? |  |
| **Q2. Drug trolleys/carts** |  |
| 2(a) How many drug trolleys are on the ward? |  |
| 2(b)(i) Is a computer device attached to the trolleys? | Yes/no/some (if some, how many) |
| 2(b)(ii) What type of computer device is attached to the trolley(s)? (e.g. built-in-computer, laptop, tablet) |  |
| 2(c)(i) Is medication stored in the trolleys in between drug rounds? (e.g. for ‘when required’ medication) | Yes/no |
| 2(c)(ii) If yes to 2(c)(i), how is medication stored in the trolleys in between drug rounds? (e.g. alphabetically by drug name, according to patient name, grouped according to type or no particular order) |  |
| 2(c)(iii) What types of medication are stored in the trolleys in between drug rounds? |  |
| 2(d) How is medication stored in the trolleys, if they are only used for storage during drug rounds? |  |
| 2(e)(i) Is medication prepared in advance for all patients for a drug round then stored on the trolley during the drug round, or prepared for each patient individually? |  |
| 2(e)(ii) Does the answer to 2(e)(ii) vary? (e.g. depending on the nurse, time of drug round) | Yes/no (If yes please specify) |
| 2(f) Who is responsible for organising medication in the trolley? |  |
| **Q3. Patient-specific medication cabinets** |  |
| 3(a) Are patient-specific medication cabinets present at the bedside? | Yes/no/some |
| 3(b) If yes to 3(a), what is their lock type? | None/digital lock/key lock/swipe card/other………..………(please specify) |
| 2(c) Who is responsible for organising medication in the patient-specific medication cabinets? |  |
| **Q4. Other storage facilities** |  |
| Q4(a) Are there any other places/ways in which medication may be kept on the ward that are not mentioned above? (not including a fridge and controlled drugs cupboard) |  |
| **Storage practices** |  |
| **Q5. Ward stock** |  |
| Q5(a) Where is general ward stock kept? |  |
| Q5(b) Where is ward stock in use by a specific patient kept? |  |
| **Q6. Patient specific medication (non-stock)** |  |
| Q6(a) Where is non-intravenous medication that has been dispensed to individual patients for inpatient use kept? |  |
| Q6(b) Where is medication that has been dispensed for discharge kept? |  |
| **Q7. Patients’ own medications** |  |
| Q7(a) Where are patients’ own medications kept while in use on the ward? |  |
| **Q8. Drug rounds** |  |
| Q8(a) How is medication transported during the drug round? |  |
| Q8(b) Where is medication for the drug round prepared? |  |
| Q8(c) Where is medication stored in between drug rounds, if prepared earlier in the day? |  |
| **Q9. Unwanted medication** |  |
| Q9(a) Where is medication for disposal stored? |  |
| **Other comments/observations** |  |
|  |  |
