# Supplementary material for: Combining research and design: A mixed methods approach aimed at understanding and optimising inpatient medication storage systems
Source: PLoS One. 2021 Dec 2;16(12):e0260197. doi: 10.1371/journal.pone.0260197 (PMC8638963; doi:10.1371/journal.pone.0260197)
Supplement: S2 Appendix — (DOCX) [file pone.0260197.s002.docx]

**S2 Appendix 2 – phase two staff and patient interview topic guides**

**Topic guide for staff interviews**

**Instructions for the researcher**

Confirm that the interviewee understands:

- The purpose of the research
- What the interview entails
- How confidentiality and anonymity will be assured
- That they can stop at any time without explanation

And that:

- They have had the chance to ask questions
- They are content to be recorded digitally (or not)

Confirm that the consent form is signed

**Topic areas**

- Confirm that the participant is a healthcare professional involved in inpatient medication administration
- Ask which specialty they work in
- Ask in what ways they are involved in the medication administration process
- What medication storage is present on the ward and what is stored where?
- What other types of health care professionals are involved in the medication administration process on this ward? Probe satisfaction with involvement/roles
- Other than physical storage and other healthcare professionals – any other factors that affect medication administration? E.g. changes when patient records went electronic
- Ask if they have experienced any issues or challenges associated with the medication administration process – probe storage, which type of medication presents the most challenges re storage e.g general floor stock, patients’ own
- Does anything work well in the current medication administration process?
- Ask if any interventions to improve medication administration – probe storage – have been proposed or implemented. How were these received? Did they result in any changes in practice?
- Are there any specific needs or requirements on this ward/in this specialty? E.g. 1:1 nursing, patients on lots of medications, physical size of the ward
- What is needed to enhance medication administration that is not currently present?
- If the ward does not have automated medication storage (e.g. Omnicell), ask whether they have experience elsewhere working with this or what their perceptions are and the potential value; pros and cons if they do have automated storage
- What would medication administration and storage look like if all medication was obtained through an individual patient dispensing system? (i.e. no ward stock) Benefits, challenges, practicalities
- What would medication administration and storage look like if all medication was supplied as floor stock? (i.e. nothing labelled for individual patients until discharge) Benefits, challenges, practicalities
- Ask if they have any suggestions for enhancing the medication administration process – probe storage
- Anything else they would like to say

**Interviewer to probe** on all answers to ensure the meaning is clear and check for understanding.

Thank participant and ask if they would like to receive a summary of the study findings and if so could they provide contact details which will be kept separate from the interview data.

**Topic guide for patient or carer interviews**

**Instructions for the researcher**

Confirm that the interviewee understands:

- The purpose of the research
- What the interview entails
- How confidentiality and anonymity will be assured
- That they can stop at any time without explanation

And that:

- They have had the chance to ask questions
- They are content to be recorded digitally (or not)

Confirm that the consent form is signed

**Topic areas**

- Confirm that the participant is/has been an inpatient (or carer of an inpatient)
- Ask them to outline the type of care they have been in receipt of without giving personal medical details e.g. inpatient for x days and basic demographic data
- Confirm that the patient has received medication whilst an inpatient and whether they have self-administered any medication
- How they found receiving medication whilst an inpatient
  - What do you expect to be happening “behind the scenes” to prepare your medication?
  - Is there anything surprising to you about how you receive medication whilst in hospital?
  - Have you observed nurses preparing medication for you? Is there anything surprising to you about this process?
- Ask if there have been any issues or challenges associated with medication administration – probe storage
- Ask if anything has worked well or what makes for a positive experience in association with medication administration
- How do you store your medication at home?
- Do all nurses seem to do the same thing?
- Ask if they have any suggestions for enhancing the medication administration process – probe storage
- Anything else they would like to say

**Interviewer to probe** on all answers to ensure the meaning is clear and check for understanding.

Thank participant and ask if they would like to receive a summary of the study findings and if so could they provide contact details which will be kept separate from the interview data.
