# Supplementary material for: Combining research and design: A mixed methods approach aimed at understanding and optimising inpatient medication storage systems
Source: PLoS One. 2021 Dec 2;16(12):e0260197. doi: 10.1371/journal.pone.0260197 (PMC8638963; doi:10.1371/journal.pone.0260197)

**S3 Appendix 3 – photographs of medication trolleys**

Type 1 – traditional drug carts with no computer: two trolleys on ward F


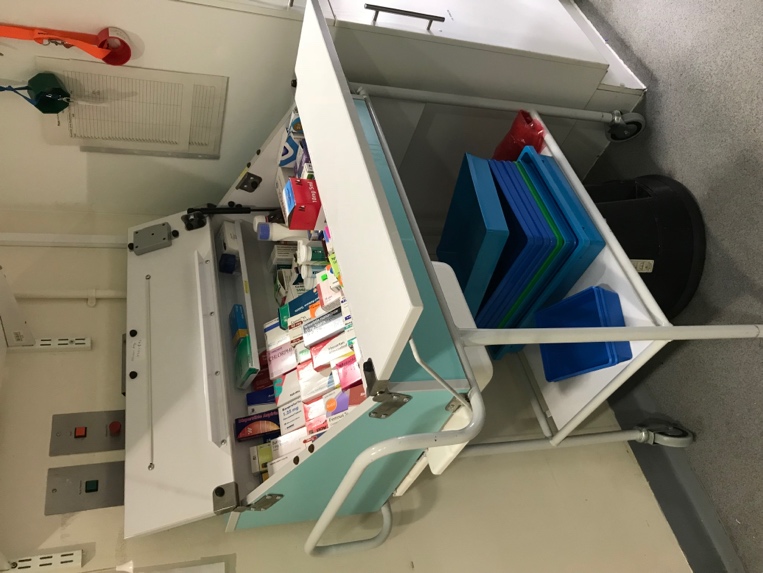


Type 2 – computers on wheels with two drawers: two trolleys on ward D and four on ward E


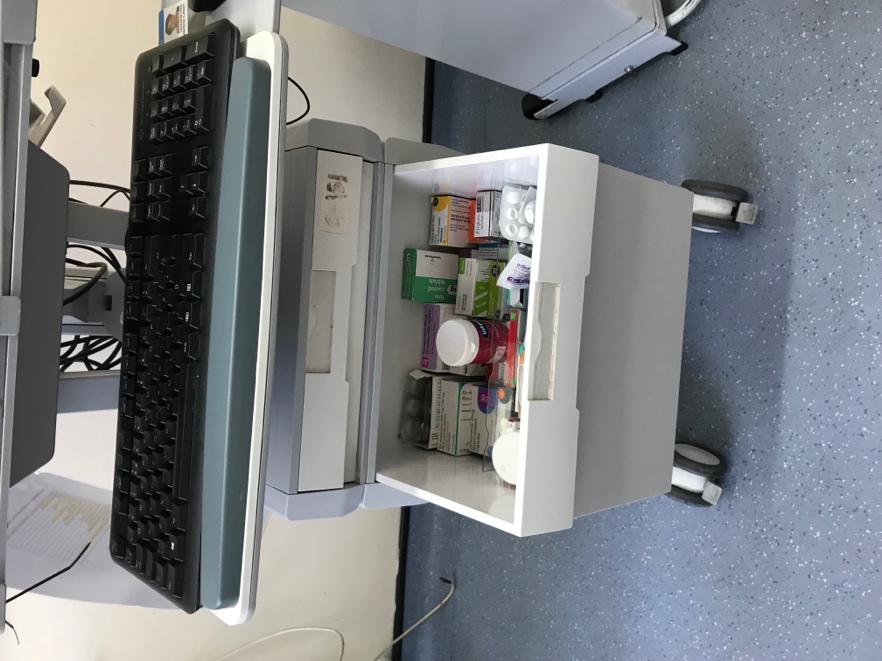


Type 3 – computers on wheels with six drawers: two trolleys on ward E.


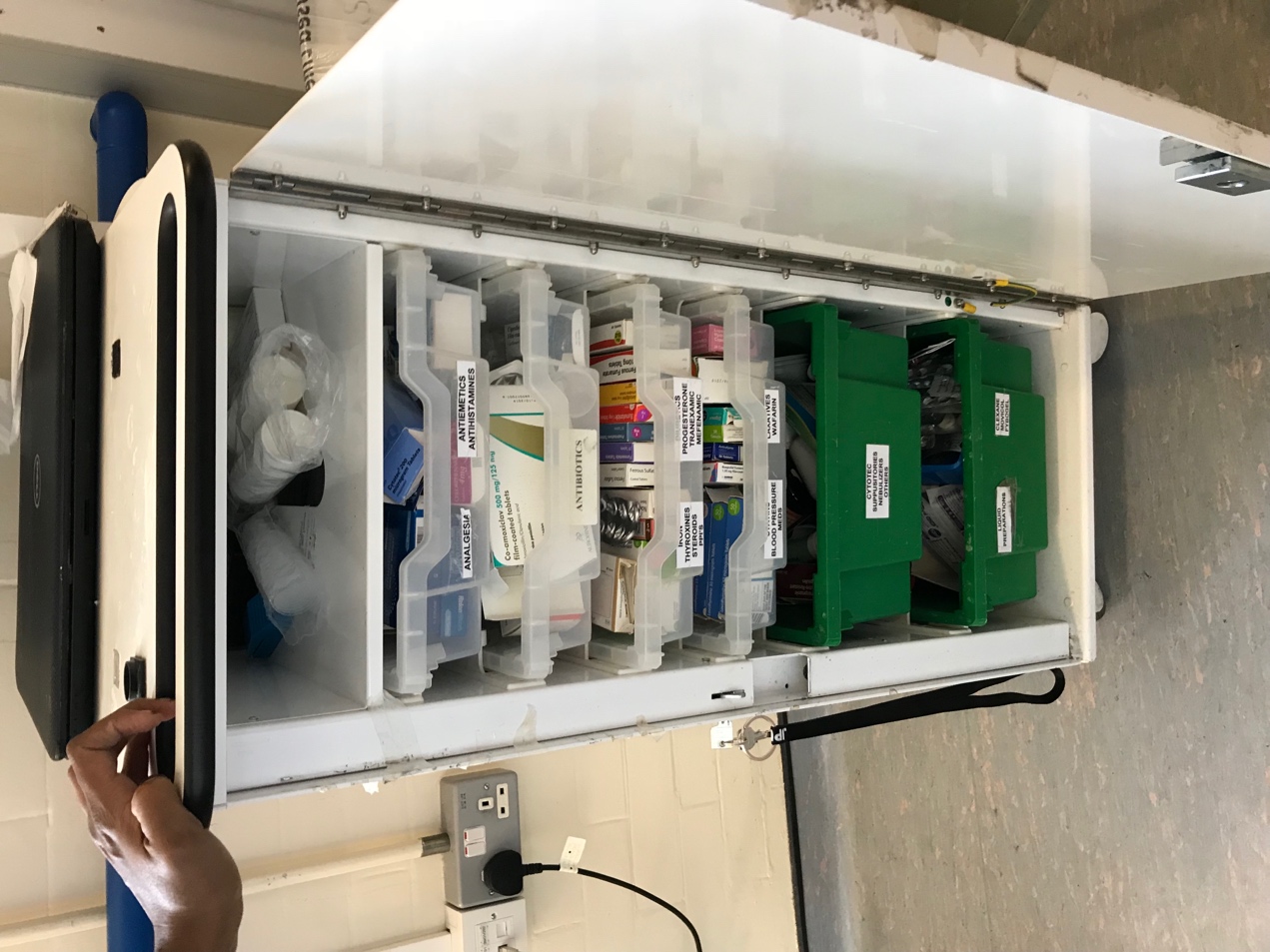

Supplement: S3 Appendix — (DOCX) [file pone.0260197.s003.docx]
