# Supplementary material for: Combining research and design: A mixed methods approach aimed at understanding and optimising inpatient medication storage systems
Source: PLoS One. 2021 Dec 2;16(12):e0260197. doi: 10.1371/journal.pone.0260197 (PMC8638963; doi:10.1371/journal.pone.0260197)
Supplement: S4 Appendix — (DOCX) [file pone.0260197.s004.docx]

#### S4 Appendix 4 – patient desires and experiences

#### Patient desires and experiences

The following themes emerged that were of importance to patients relating to the storage of medication:

#### Patients’ ability to self-administer medication

The first of these related to inpatients’ ability to self-administer medication. There was variation among the 12 patients interviewed in their desire to self-administer. Of the nine patients with long-term conditions who were administering their own medication at home, three were also doing so during their stay. Reasons for wanting to self-administer medication included maintaining a routine, having a sense of control during a time when things felt out of their control, having something to do, and not trusting staff to administer medication correctly with fears about being given the wrong dose or type of medication.

Patients’ medication for self-administration was stored in locked bedside lockers for two patients, who both knew the key code to open the locker (one code was individual to that locker; the other was a general code for all lockers on the ward). Another patient was keeping her medication in a suitcase rather than her locker; she reported that this was because there had been problems with patients forgetting the key code. Reflecting on this, the patient felt that it might not be the safest approach, should someone else come along and access her medication. A fourth participant was occasionally self-administering an injectable medication that was stored in his bedside locker, which he did not have access to, and so could only be removed by a nurse.

Patients who were not self-administering their own medication but wished to, or who were currently doing so but had not been allowed to in other settings, described frustration at not being able to access medication themselves. One patient with a long-term condition described how she had previously self-administered her own medication during hospital stays, but after the following negative experience where temporary staff were unable to find her medication, she had decided not to anymore:

*“I needed someone to go and get it [the medication] from their fridge. I’m not allowed to go there. But sometimes there are a few fridges and they can’t find [it]. So that’s why I didn’t bring anything with me this time and that’s much easier, calmer.”* (Patient 2, Ward B)

Another long-term inpatient described how she was keeping the medication she was currently using on her person, separate to the large quantity of medication in her bedside locker that needed to be removed:

*“I’m actually currently living out of a freezer bag, with one repeat of what I’m taking. I seem to have been…over provided. So I’ve got what I would call a bit of an overflow here…loads.”* (Patient 1, Ward D)

Conversely, several patients gave reasons why they did not feel self-administration was appropriate. These included the risks of other patients having access to their medication, a general view that only staff should be able to access medication and concerns over patients not always being mentally competent. Additionally, some patients felt they were too unwell, overwhelmed during their current stay and/or had changes in the medication that they were currently taking.

*“I think it’s a difficult one, because when you’re not feeling well you might forget. And if you have something happen that you can’t cope with and it gets so busy and hectic…but they [nurses] might not remember because they get so rushed off their feet and then they wouldn’t do it and you’ll be waiting again. I think it’s probably good for them to do it, because there’s so many things going on, it’s a nightmare. (Patient 2, Ward E)*

*“The thing is I’ve had so much stuff, by the time you get in here to where I am, it’s like they know what they’re doing, just let them get on with it and just take the medicine” Obviously they’ve got to have a level of control over it, so they can’t just give you boxes of the stuff and leave it down to yourself” (Patient 2, Ward C).*

#### Patients’ desire for information

Secondly, patients overwhelmingly expressed a desire to know what medication they were receiving. While most patients reported trusting hospital staff (although not universally), they also described being an active participant in their medication process, checking, querying and trying to understand what they were being given. When asked whether he knew where his medication was stored and prepared, one patient replied:

*“No idea. Wish I did. Just to see, just to make sure you get the right dose.”* (Patient 2, Ward D).

As well as wanting to be able to see their medication being prepared, patients also wanted to receive information about their medication and to be able to use the opportunity of a medication round to ask questions to nursing staff. Several patients wondered whether they might be able to have a chart or app that would provide information on their medication and upcoming doses.

*“Most people have got smart phones now, so if there was a programme that you could log into and go oh, at two o’clock I’m having that and at six o’clock I’m having that”* (Patient 2, Ward C)

#### Receiving medication when it is due

Thirdly, when asked about whether they had been satisfied with their experience of receiving medication, almost all patients linked a good experience to one where their medication is given on time:

*“It’s been good on this ward, this time specifically it’s been on time. Especially once the nurses get to know you and your medication, they’re always on time.”* (Patient 2, Ward B)

This was true for all patients but particularly emphasized by those who were receiving medication for a condition where the regularity and timing of doses of medication was particularly important and by those receiving analgesia. Around half of those interviewed recollected at least one incident where they had been left waiting or without their medication.
