# Supplementary figures and images for: Combining research and design: A mixed methods approach aimed at understanding and optimising inpatient medication storage systems
Source: PLoS One. 2021 Dec 2;16(12):e0260197. doi: 10.1371/journal.pone.0260197 (PMC8638963; doi:10.1371/journal.pone.0260197)

**S5 Appendix 5 – prototype solution**

**
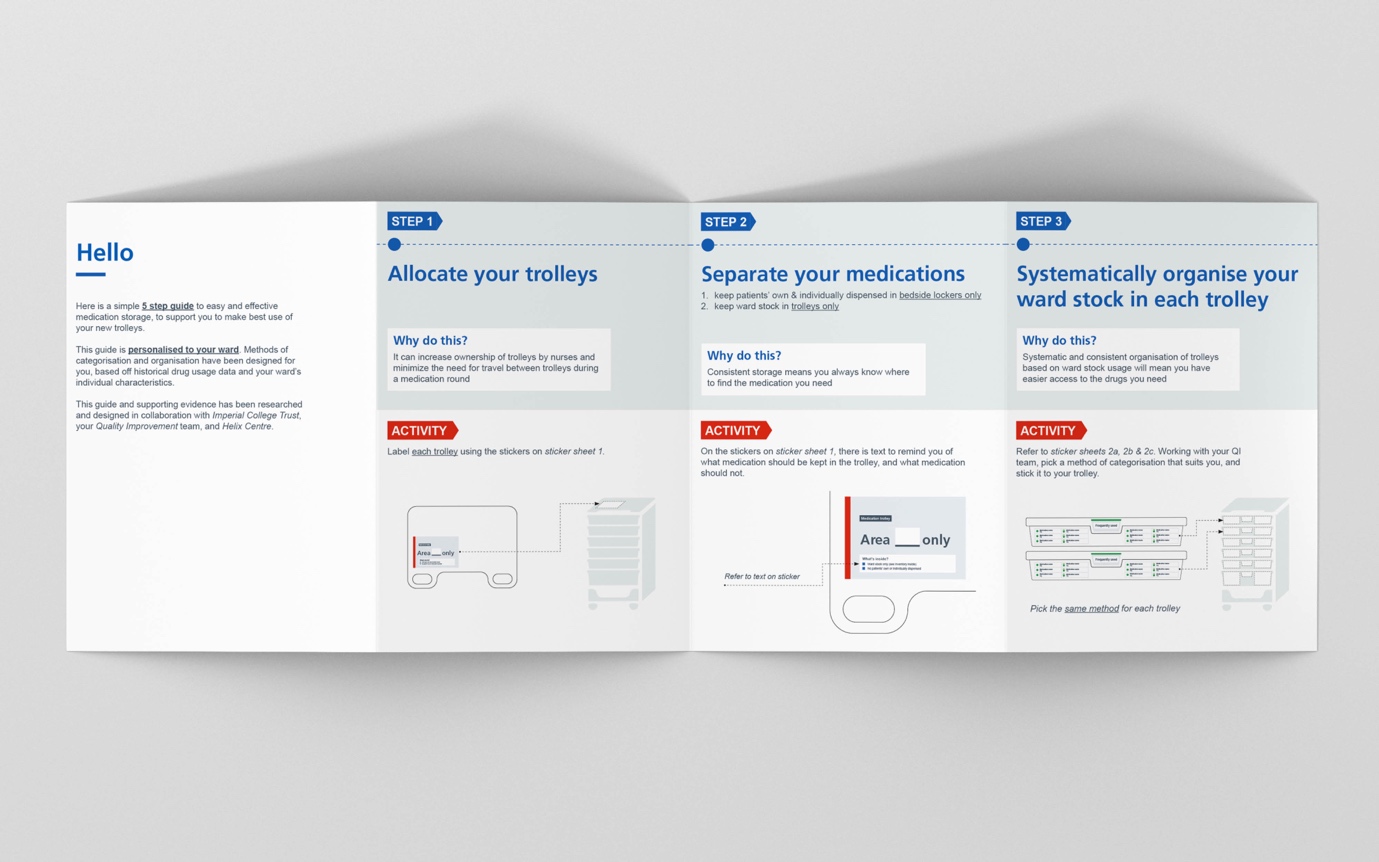
**

**
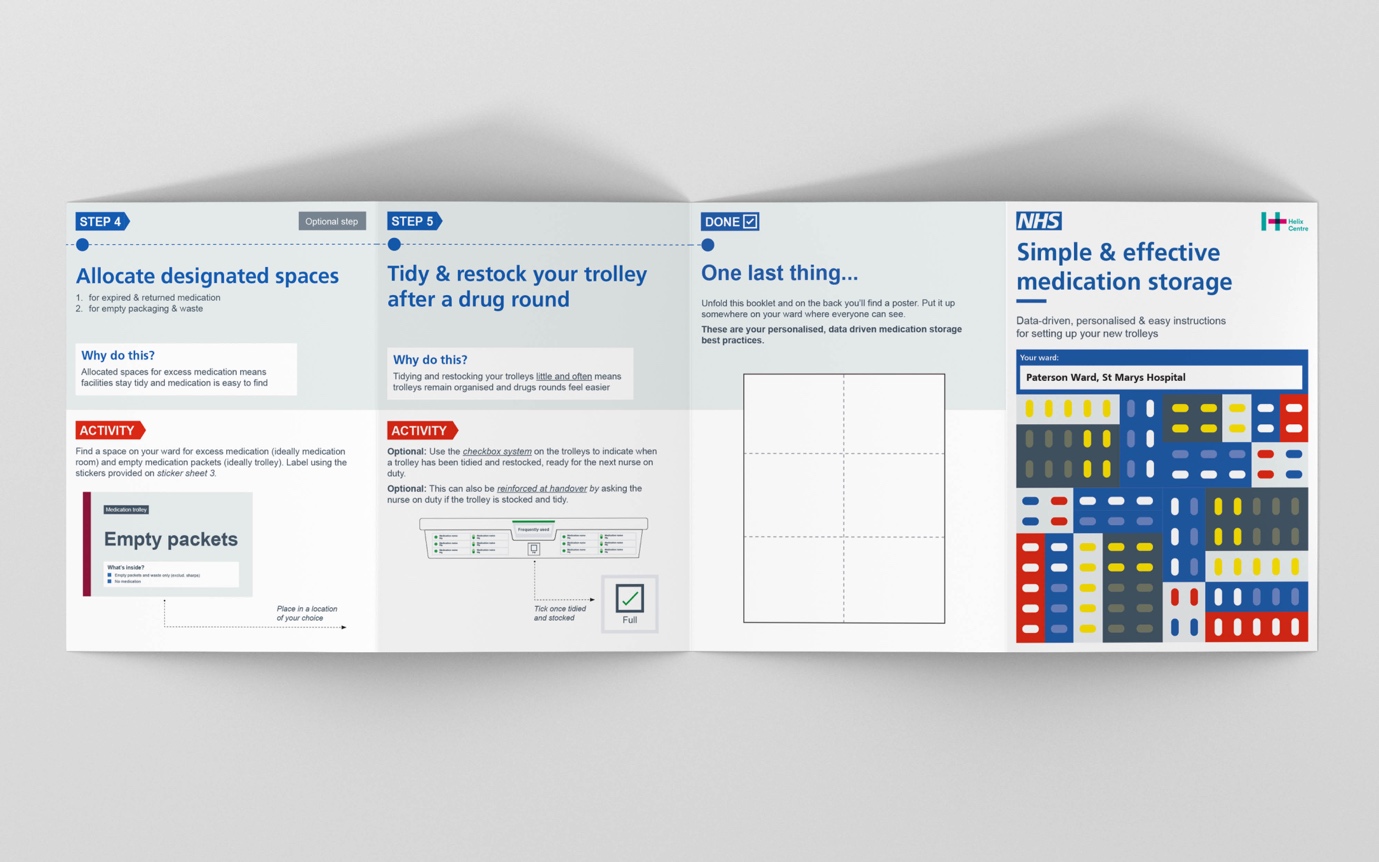
**

**
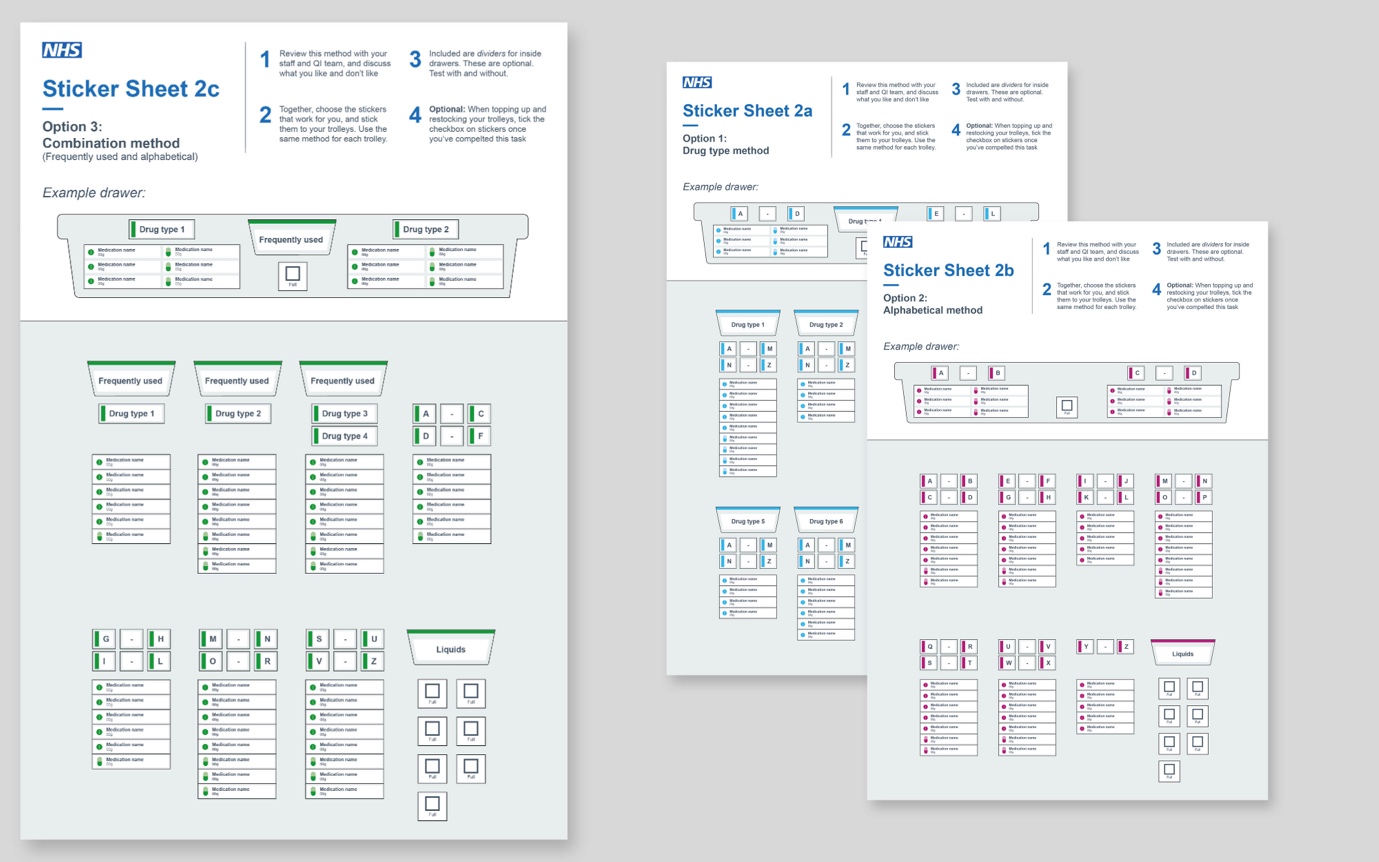
**

Supplement: S5 Appendix — (DOCX) [file pone.0260197.s005.docx]
