# Supplementary material for: Combining research and design: A mixed methods approach aimed at understanding and optimising inpatient medication storage systems
Source: PLoS One. 2021 Dec 2;16(12):e0260197. doi: 10.1371/journal.pone.0260197 (PMC8638963; doi:10.1371/journal.pone.0260197)
Supplement: S6 Appendix — (DOCX) [file pone.0260197.s006.docx]

**S6 Appendix 6 – anonymised survey data from phase one**

**Table A1. Number of beds on wards at hospital sites 1-4**

|  | **Hospital site 1** | **Hospital site 2** | **Hospital site 3** | **Hospital site 4** |
| --- | --- | --- | --- | --- |
| **Ward** | **No. of inpatient beds** | **No. of inpatient beds** | **No. of inpatient beds** | **No. of inpatient beds** |
| **1** | 21 | 16 | 15 | 26 |
| **2** | 15 | 31 | 17 | 15 |
| **3** | 32 | 12 | 15 | 20 |
| **4** | 26 | 21 | 17 | 12 |
| **5** | 23 | 28 | 8 | 21 |
| **6** | 21 | 21 | 12 | 15 |
| **7** | 24 | 13 | 8 | 7 |
| **8** | 19 | 16 | 17 | 8 |
| **9** | 15 | 27 | 16 | - |
| **10** | 5 | 24 | 20 | - |
| **11** | 23 | 20 | 12 | - |
| **12** | 25 | 20 | 5 | - |
| **13** | 13 | 9 | 11 | - |
| **14** | 18 | 16 | 25 | - |
| **15** | 23 | 14 | 23 | - |
| **16** | 19 | 15 | 19 | - |
| **17** | 13 | 25 | 16 | - |
| **18** | 26 | - | 16 | - |
| **19** | 25 | - | 30 | - |
| **20** | 24 | - | 14 | - |
| **21** | 14 | - | 28 | - |
| **22** | 13 | - | 12 | - |
| **23** | 22 | - | 11 | - |
| **24** | 20 | - | 35 | - |
| **25** | **-** | **-** | 22 | - |
| **26** | **-** | **-** | 9 | - |
| **27** | **-** | **-** | 20 | - |
| **28** | **-** | **-** | 10 | - |
| **Total no. beds** | **479** | **328** | **463** | **124** |

**Table A2. Medication rooms at hospital site 1**

| **Ward** | **Approx. Size**  **(m^2^)** | **Location** | **Room lock type** | **Medication cupboards lockable?** | **Responsible for organising medication** | **Responsible for unpacking medication** |
| --- | --- | --- | --- | --- | --- | --- |
| 1 | 24 | Beside nurses’ station | Digital | Yes | Nursing staff | Nursing staff |
| 2 | 10 | Beside nurses’ station | Digital | No | Nursing staff | Nursing staff |
| 3 | 6, 7 | Beside nurses’ station | Digital | Yes | Nursing staff | Nursing staff and health care assistants |
| 4 | 9 | Beside nurses’ station | Digital | Some | Nursing staff | Nursing staff |
| 5 | 14 | Beside nurses’ station | Digital | Yes | Pharmacy staff | Nursing staff |
| 6 | 24 | Beside nurses’ station | Digital | No | Nursing and pharmacy staff | Nursing staff |
| 7 | 18 | Beside nurses’ station | Digital | Yes | Nursing staff | Nursing staff |
| 8 | 14 | Beside nurses’ station | Digital | Yes | Nursing and pharmacy staff | Nursing staff |
| 9 | 11 | Beside nurses’ station | Digital | Yes | Nursing staff | Nursing staff |
| 10 | 15 | Beside nurses’ station | Digital | Yes | Nursing staff | Nursing staff |
| 11 | 14 | Beside nurses’ station | Digital | No | Nursing staff | Nursing staff |
| 12 | 16 | Beside nurses’ station | Digital | Yes | Nursing staff | Nursing staff |
| 13 | 17 | Around the corner from nurses’ station – into unit | Digital | No | Nursing staff | Nursing staff |
| 14 | 5 | Around corner from nurses’ station into unit | Digital | No | Nursing staff | Nursing staff |
| 15 | 8 | Beside nurses’ station | Digital | Yes | Nursing staff | Nursing staff |
| 16 | 16 | Beside nurses’ station | Digital | Yes | Nursing and pharmacy staff | Nursing staff |
| 17 | 10 | Beside nurses’ station | Digital | Yes; and automated dispensing cabinet | Automated dispensing cabinet – pharmacy staff, other – nursing | Nursing staff |
| 18 | 13 | Beside nurses’ station | Swipe card | Yes; and automated dispensing cabinet | Pharmacy staff | Pharmacy staff |
| 19 | 14 | Beside nurses’ station | Digital | No | Nursing and pharmacy staff | Nursing staff |
| 20 | 6 | Beside nurses’ station | Digital | No | Nursing and pharmacy staff | Nursing staff |
| 21 | 5 | Beside reception desk in the middle of the unit | Swipe card | Yes | Nursing staff | Nursing staff |
| 22 | 12 | Lobby before entering the unit | Swipe card | No | Nursing staff | Nursing staff and health care assistants |
| 23 | 12 | Beside nurses’ station | Digital | Yes | Nursing and pharmacy staff | Nursing staff |
| 24 | 6 | Down corridor from nurse's station on one side of the unit | Digital | Yes | Nursing staff | Nursing staff |

**Table A3. Medication rooms at hospital site 2**

| **Ward** | **Approx. size (m^2^)** | **Location** | **Room lock type** | **Medication cupboards lockable?** | **Responsible for organising medication** | **Responsible for unpacking medication** |
| --- | --- | --- | --- | --- | --- | --- |
| 1 | 8 | At one end of the ward | Digital | Yes | Nursing staff | Nursing staff |
| 2 | 12 | Down the corridor from nurses’ station - at entrance to ward | Digital | Yes | Nursing staff | Nursing staff |
| 3 | 15 | At one end of the ward | Digital | Yes | Pharmacy staff | Pharmacy staff |
| 4 | 12 | Down the corridor from nurses’’ station, on the way into unit | Digital | No | Nursing and pharmacy staff | Nursing and pharmacy staff |
| 5 | 10 | Beside nurses’ station | Digital | Yes | Nursing and pharmacy staff | Nursing and pharmacy staff |
| 6 | 12 | Down the corridor from nurse’s station | Digital | Yes | Nursing staff | Nursing staff |
| 7 | 10 | Beside nurses’ station | Key | Yes | Nursing staff | Nursing staff |
| 9 | 5 | Beside nurses’ station | Digital | Yes | Nursing staff | Nursing staff |
| 10 | 16 | Beside nurses’ station | Digital | Yes | Nursing staff | Nursing staff |
| 11 | 16 | Between bays - at end of the ward | Digital | Some | Nursing staff | Nursing staff |
| 12 | 15 | End of the ward | Digital | Yes | Nursing staff | Nursing staff |
| 13 | 6 | Around the corner from nurses’ station – into unit | Digital | No | Nursing staff | Nursing staff |
| 14 | 8 | Around the corner from nurses’ station | Digital | Yes | Nursing and pharmacy staff | Nursing and pharmacy staff |
| 15 | 16 | Into the unit and at one end | Digital | Yes | Nursing staff | Nursing staff |
| 16 | 11 | Down the corridor from nurses’ station at one side of the ward | Digital | Yes | Nursing staff | Nursing staff |
| 17 | 12 | At the end of a corridor | Digital | Yes | Nursing staff and students; health care assistants | Nursing staff and students; health care assistants |

**Table A4. Medication rooms at hospital site 3**

| **Ward** | **Approx. size**  **(m^2^)** | **Location** | **Room lock** | **Medication cupboards lockable?** | **Responsible for organising medication** | **Responsible for unpacking medication** |
| --- | --- | --- | --- | --- | --- | --- |
| 1 | 15 | Down the corridor from nurses’ station | Digital | Yes | Nursing staff | Nursing staff |
| 2 | 12 | Beside nurses’ station | RFID | Yes | Nursing staff | Nursing staff |
| 3 | 10 | In one of the bays | Digital | Yes | Nursing staff | Nursing staff |
| 4 | 8 | Between two nurses’ stations | Digital | Yes | Nursing staff | Nursing staff |
| 5 | 12 | At one end of the ward | Digital | Yes | Nursing and pharmacy staff | Nursing staff |
| 6 | 11 | To one side of ward and round corner from nurses’ station | Digital | Yes | Nursing staff | Nursing staff |
| 7 | 7 | Beside nurses’ station | Digital | No | Nursing staff | Nursing staff and health care assistants |
| 8 | 17 | At entrance to ward across from nurses’ station | Swipe card | No | Nursing and pharmacy staff | Nursing staff |
| 9 | 8 | Down the corridor from nurses’ station | Digital | Yes | Nursing staff | Nursing staff |
| 10 | 9 | In the middle of ward, with nurses’ stations at each end | Digital | Yes | Nursing staff | Nursing staff |
| 12 | 11 | Down corridor on one side from nurses’ station | Digital | Yes | Nursing staff | Nursing staff |
| 14 | 10 | Beside nurses’ station | Digital | Yes | Nursing staff | Nursing staff |
| 15 | 17 | Beside nurses’ station | Swipe card | Some | Nursing staff | Nursing staff |
| 16 | 5 | Around the corner from nurses’ station | Swipe card | No | Nursing staff | Nursing staff |
| 17 | 3, 6 | One each side of unit and close to nurses’ stations | Swipe card | No | Nursing staff | Nursing staff |
| 18 | 11 | Beside nurses’ station | Swipe card | Some | Nursing staff | Nursing staff |
| 19 | 5 | Beside nurses’ station | Digital | Yes | Nursing and pharmacy staff | Nursing staff |
| 20 | 10 | Beside nurses’ station | Swipe card | Yes | Nursing staff | Nursing staff |
| 21 | 14 | Beside nurses’ station | Digital | Some | Nursing staff | Nursing staff |
| 23 | 5 | Down the corridor from nurses’ station | Swipe card | Yes | Nursing staff | Nursing staff |
| 24 | 4 | Twp nurses’ stations at each end of ward medication room in the middle | Swipe card | No | Nursing staff | Midwifery and nursing staff |
| 25 | 80 | Down the corridor from nurses’ station | None | Yes | Nursing staff | Nursing staff |
| 26 | 22 | Down the corridor from nurses’ station – end of unit | Swipe card | Yes | Nursing and pharmacy staff | Nursing staff |
| 27 | 12 | Beside nurses’ station | Swipe card | No | Nursing and pharmacy staff | Nursing staff and housekeepers |

**Table A5. Medication rooms at hospital site 4**

| **Ward** | **Approx. size**  **(m^2^)** | **Location** | **Room lock** | **Medication cupboards lockable?** | **Responsible for organising medication** | **Responsible for unpacking medication** |
| --- | --- | --- | --- | --- | --- | --- |
| 1 | 9 | Beside nurses’ station | Swipe card | Yes | Nursing staff | Nursing staff |
| 2 | 7 | Beside nurses’ station | Swipe card | Yes | Nursing staff | Nursing staff |
| 3 | 8 | Beside nurses’ station | Swipe card | Yes | Nursing staff | Nursing staff and health care assistants |
| 4 | 7 | Beside nurses’ station | Swipe card | Yes | Midwifery staff | Midwifery staff |
| 5 | 9, 7 | Two - opposite ends of the ward | Swipe card | No | Midwifery and nursing staff | Midwifery and nursing staff |
| 6 | Unknown | At one end of unit within bay close to nurse’s station | Unknown | Some | Nursing staff | Nursing staff |
| 7 | 8 | Down the corridor from the nurses’ station | Digital | No | Nursing staff | Nursing staff |
| 8 | 9 | Beside nurses’ station | Digital | Yes | Midwifery staff | Maternity support worker |

**Table A6. Medication trolleys at hospital site 1**

| **Ward** | **No. trolleys** | **Computer device attached?** | **Medication stored between drug rounds?** | **Medication arrangement** | **Type of medication stored** | **How is medication prepared during the drug round?** | **Responsible for organising the medication** |
| --- | --- | --- | --- | --- | --- | --- | --- |
| 1 | 3 | Yes | Yes | Alphabetically | Most frequently used ward stock | Individually | Nursing staff |
| 3 | 1 | No | Yes | Alphabetically | Most frequently used ward stock | Individually | Nursing staff |
| 6 | 3 | Yes | Yes | Alphabetically | Most frequently used ward stock | Individually | Nursing staff |
| 8 | 1 | No | Yes | Alphabetically | Most frequently used ward stock | Individually | Nursing staff |
| 9 | 2 | Yes | Yes | No order | Most frequently used ward stock | Individually | Nursing staff |
| 12 | 2 | No | Yes | Alphabetically | Most frequently used ward stock | Individually | Nursing staff |
| 13 | 2 | No | Yes | Alphabetically and by type | Most frequently used ward stock | Individually | Nursing staff |
| 14 | 2 | No | Yes | Alphabetically | All stock | Individually | Nursing staff |
| 15 | 3 | Yes | Yes | Alphabetically | Most frequently used ward stock | Individually | Nursing staff |

**Table A7. Medication trolleys at hospital site 2**

| **Ward** | **No. trolleys** | **Computer device attached?** | **Medication stored between drug rounds?** | **Medication arrangement** | **Type of medication stored** | **How is medication prepared during the drug round?** | **Responsible for organising the medication** |
| --- | --- | --- | --- | --- | --- | --- | --- |
| 1 | 2 | Yes | Yes | Alphabetically | Most frequently used ward stock | Individually | Nursing staff |
| 7 | 1 | No | Yes | Alphabetically | Most frequently used ward stock | Individually | Nursing staff |
| 10 | 5 | Yes | Yes | Varies with different nursing staff | Most frequently used ward stock | Individually | Nursing staff |
| 12 | 1 | No | Yes | By type | Most frequently used ward stock | Individually | Nursing staff |

**N.B. Two wards also reported having medication trolleys but were not using them**

**Table A8. Medication trolleys at hospital site 3**

| **Ward** | **No. trolleys** | **Computer device attached?** | **Medication stored between drug rounds?** | **Medication arrangement** | **Type of medication stored** | **How is medication prepared during the drug round?** | **Responsible for organising the medication** |
| --- | --- | --- | --- | --- | --- | --- | --- |
| 1 | 2 | Yes | Yes | So as is visible | Most frequently used ward stock | Individually | Nursing staff |
| 2 | 3 | 2 x Yes; 1 x No | Yes | Alphabetically, type | Most frequently used ward stock | Individually | Nursing staff |
| 3 | 2 | Yes | Yes | No order | Most frequently used ward stock | Individually | Nursing staff |
| 4 | 2 | No | Yes | By type | Most frequently used ward stock | Individually | Nursing staff |
| 5 | 2 | Yes | Yes | No order | Most frequently used ward stock | Individually | Nursing staff |
| 6 | 2 | Yes | Yes | Alphabetically | Most frequently used ward stock | Individually | Nursing staff |
| 7 | 2 | Yes | Yes | No order | Most frequently used ward stock | Individually | Nursing staff |
| 8 | 4 | Yes | Yes | No order | Most frequently used ward stock | Individually | Nursing staff |
| 9 | 5 | Yes | Yes | By type | Most frequently used ward stock | Individually | Nursing staff |
| 10 | 4 | Yes | Yes | Alphabetically | Most frequently used ward stock | Individually | Nursing staff |
| 13 | 1 | Yes | No | No order | Most frequently used ward stock | Altogether | Nursing staff |
| 14 | 4 | No | Yes | Alphabetically; by type | Most frequently used ward stock | Individually | Nursing staff |
| 15 | 3 | Yes | Yes | Alphabetically | Most frequently used ward stock | Individually | Nursing staff |
| 19 | 3 | Yes | Yes | By type | Most frequently used ward stock | Individually | Nursing staff |
| 20 | 2 | No | Yes | So as is visible | Most frequently used ward stock | Individually | Nursing staff |
| 21 | 3 | Yes | Yes | Alphabetically | Most frequently used ward stock | Individually | Nursing staff |
| 22 | 1 | Yes | Yes | Alphabetically | Most frequently used ward stock | Individually | Nursing staff |
| 24 | 3 | Yes | Yes | So as is visible | Most frequently used ward stock | Individually | Midwifery staff |
| 25 | 1 | No | Yes | So as is visible | Most frequently used ward stock | Individually | Nursing staff |

**Table A9. Medication trolleys at hospital site 4**

| **Ward** | **No. trolleys** | **Computer device attached?** | **Medication stored between drug rounds?** | **Medication arrangement** | **Type of medication stored** | **How is medication prepared during the drug round?** | **Responsible for organising the medication** |
| --- | --- | --- | --- | --- | --- | --- | --- |
| 1 | 2 | Yes | Yes | By type | Most frequently used ward stock | Individually | Nursing staff |
| 2 | 1 | Yes | Yes | By type | Most frequently used ward stock | Individually | Midwifery staff |
| 3 | 1 | No | Yes | No order | Most frequently used ward stock | Individually | Nursing staff |
| 4 | 1 | Yes | Yes | So as is visible | Most frequently used ward stock | Individually | Midwifery staff |
| 6 | 1 | No | Yes | By type | Most frequently used ward stock | Individually | Nursing staff |
| 7 | 1 | No | Yes | By type | Most frequently used ward stock | Individually | Nursing staff |
| 8 | 1 | Yes | Yes | By type | Most frequently used ward stock | Individually | Midwifery staff |

**Table A10. Bedside medication storage at hospital site 1**

| **Ward** | **Lock type** | **Responsible for organising** |
| --- | --- | --- |
| 1 | Digital and key | Nursing staff |
| 2 | Key | Nursing staff |
| 3 | Digital | Nursing staff |
| 4 | Key | Nursing staff |
| 5 | Digital and key | Nursing staff |
| 6 | Key | Nursing staff |
| 7 | Key | Nursing staff |
| 8 | Key | Nursing staff |
| 9 | Key | Nursing staff |
| 10 | Digital | Nursing staff |
| 11 | Key | Nursing staff |
| 12 | Key | Nursing staff |
| 13 | Key | Nursing staff |
| 14 | Key | Nursing staff |
| 15 | Key | Nursing staff |
| 16 | Digital | Nursing staff |
| 17 | Key | Nursing staff |
| 18 | Digital | Nursing staff |
| 19 | Key | Nursing staff |
| 20 | Digital and key | Nursing staff |
| 21 | Digital | Nursing staff |
| 22 | Digital | Nursing staff |
| 23 | Digital | Nursing staff |
| 24 | Key | Nursing staff |

**Table A11. Bedside medication storage at hospital site 2**

| **Ward** | **Lock type** | **Responsible for organising** |
| --- | --- | --- |
| 1 | Digital and key | Nursing staff |
| 2 | Digital | Nursing staff |
| 3 | Key | Nursing and pharmacy staff |
| 4 | Digital | Nursing staff |
| 5 | Key | Nursing staff |
| 6 | Key | Nursing staff |
| 7 | Key | Nursing staff |
| 8 | Key | Nursing staff |
| 9 | Key | Nursing and pharmacy staff |
| 10 | Key | Nursing staff |
| 11 | Key | Nursing staff |
| 12 | Key | Nursing staff |
| 13 | Combination padlock | Nursing staff |
| 14 | Key | Nursing staff |
| 16 | Key | Nursing staff |
| 17 | Key | Nursing staff |

**Table A12. Bedside medication storage at hospital site 3**

| **Ward** | **Lock type** | **Responsible for organising** |
| --- | --- | --- |
| 1 | Digital | Nursing staff |
| 2 | Key | Nursing staff |
| 3 | Digital | Nursing staff |
| 4 | Digital | Nursing staff |
| 5 | Digital | Nursing staff |
| 6 | Combination padlock | Nursing staff |
| 8 | Digital | Nursing staff |
| 9 | Digital | Nursing staff |
| 10 | Digital | Nursing and pharmacy staff |
| 11 | Key | Nursing staff |
| 14 | Key | Nursing staff |
| 15 | Digital | Nursing staff |
| 17 | Digital | Nursing staff |
| 18 | Key | Nursing staff |
| 19 | Digital | Nursing staff |
| 20 | Digital | Nursing staff |
| 21 | Digital and key | Nursing staff |
| 22 | Key | Nursing staff |
| 23 | Key | Nursing staff |

**Table A13. Bedside medication storage at hospital site 4**

| **Ward** | **Lock type** | **Responsible for organising** |
| --- | --- | --- |
| 3 | Key | Nursing staff |

**Table A14. Medication storage practices at hospital site 1**

| **Ward** | **Ward stock** | **Ward stock in use by a specific patient** | **Medication dispensed to patients for inpatient use** | **Patients’ own medication** | **Medication dispensed for discharge** | **Medication for returning to pharmacy** |
| --- | --- | --- | --- | --- | --- | --- |
| 1 | Medication room, trolley | Trolley | Bedside, or if too big in the medication room | Bedside | Medication room | Medication room |
| 2 | Medication room | Bedside | Bedside | Bedside | Medication room | Medication room |
| 3 | Medication room, trolley | Medication room, trolley | Medication room, trolley | Bedside | Bedside | Given to pharmacy |
| 4 | Medication room | Bedside | Bedside | Bedside | Medication room | Medication room |
| 5 | Medication room | Bedside | Bedside | Bedside | Medication room, bedside | Medication room |
| 6 | Medication room, trolley | Trolley | Bedside | Bedside | Medication room | Medication room |
| 7 | Medication room | Medication room | Bedside | Bedside | Medication room | Medication room |
| 8 | Medication room, trolley | Trolley | Bedside | Bedside | Medication room | Medication room |
| 9 | Medication room, trolley | Medication room, trolley | Bedside | Bedside | Medication room or if small maybe at the bedside | Medication room |
| 10 | Medication room | Medication room | Bedside | Bedside | Medication room | Medication room |
| 11 | Medication room | Medication room | Bedside | Not stored | Medication room | Medication room |
| 12 | Medication room, trolley | Medication room, trolley | Bedside | Bedside | Cupboard outside of medication room by nurses’ station | Medication room |
| 13 | Medication room, trolley | Medication room, trolley | Bedside | Bedside | Bedside or if lock broken in the medication room | Medication room |
| 14 | Medication room, trolley | Bedside, trolley | Bedside, or if too big in the medication room | Bedside | Cupboard external to the medication room | Medication room |
| 15 | Medication room, trolley | Trolley | Bedside | Bedside | Medication room | Medication room |
| 16 | Medication room | Medication room | Bedside | Bedside | Medication room, bedside | Medication room |
| 17 | Medication room – automated dispensing cabinet | Bedside | Bedside | Bedside | Cupboard on nearby ward | Medication room |
| 18 | Medication room – automated dispensing cabinet | Bedside | Bedside | Bedside | Medication room | Medication room |
| 19 | Medication room | Bedside | Bedside | Bedside | Medication room | Medication room |
| 20 | Medication room | Medication room | Bedside | Bedside | Bedside | Medication room |
| 21 | Medication room | Medication room, bedside | Bedside | Bedside | Bedside | Pharmacy remove |
| 22 | Medication room | Bedside | Bedside | Bedside | Not stored | Pharmacy remove |
| 23 | Medication room | Bedside | Bedside | Bedside | Medication room, bedside | Medication room |
| 24 | Medication room | Medication room | Bedside | Bedside | Medication room | Nurses’ station |

**Table A15. Medication storage practices at hospital site 2**

| **Ward** | **Ward stock** | **Ward stock in use by a specific patient** | **Medication dispensed to patients for inpatient use** | **Patients’ own medication** | **Medication dispensed for discharge** | **Medication for returning to pharmacy** |
| --- | --- | --- | --- | --- | --- | --- |
| 1 | Medication room, trolley | Medication room, trolley | Bedside | Bedside | Bedside | Medication room |
| 2 | Medication room | Medication room | Bedside | Bedside | Bedside | Medication room |
| 3 | Medication room | Medication room | Medication room | Bedside | Bedside | Medication room |
| 4 | Medication room | Medication room | Bedside | Bedside | Bedside | Medication room |
| 5 | Medication room | Medication room | Bedside | Bedside | Bedside | Medication room |
| 6 | Medication room | Medication room | Bedside | Bedside | Bedside | Medication room |
| 7 | Medication room | Medication room | Bedside | Bedside | Bedside | Medication room |
| 8 | Medication cupboard | Bedside | Bedside | Bedside | Not stored | Box at end of ward |
| 9 | Medication room | Medication room | Bedside | Bedside | Bedside | Medication room |
| 10 | Medication room, trolley | Trolley | Bedside | Bedside | Bedside | Medication room |
| 11 | Medication room | Bedside | Bedside | Bedside | Bedside | Medication room |
| 12 | Medication room, trolley | Trolley | Bedside | Bedside | None stored | Medication room |
| 13 | Medication room | Medication room | Bedside | Bedside | Bedside locker if name on from pharmacy; have some ward stock they give and that is kept in medication room | Medication room |
| 14 | Medication room | Bedside | Bedside | Bedside | Not stored | Medication room |
| 15 | Medication room | Medication room | Medication room | Medication room | Medication room | Medication room |
| 16 | Medication room | Bedside | Bedside | Bedside | Medication room | Medication room |
| 17 | Medication room | Bedside | Bedside | Bedside | Fluid room | Medication room |

**Table A16. Medication storage practices at hospital site 3**

| **Ward** | **Ward stock** | **Ward stock in use by a specific patient** | **Medication dispensed to patients for inpatient use** | **Patients’ own medication** | **Medication dispensed for discharge** | **Medication for returning to pharmacy** |
| --- | --- | --- | --- | --- | --- | --- |
| 1 | Medication cupboard, trolley | Trolley | Bedside | Bedside | Bedside | Medication cupboard |
| 2 | Medication room, trolley | Trolley | Bedside | Bedside | Medication room, bedside | Medication room |
| 3 | Medication room, trolley | Trolley | Bedside | Bedside | Medication room | Medication room |
| 4 | Medication room, trolley | Trolley | Bedside | Bedside, but side rooms don’t have a locker so overspill in medication room | Medication room | Medication room |
| 5 | Medication room, trolley | Trolley | Bedside | Bedside | Bedside, medication room | Medication room |
| 6 | Medication room, trolley | Trolley | Bedside | Medication room, bedside | Medication room | Medication room |
| 7 | Medication room, trolley | Medication room, trolley | Drawer with number in medication room | Medication room | Medication room | Medication room |
| 8 | Medication room, trolley | Medication room, trolley | Bedside | Bedside | Bedside | Pharmacy remove |
| 9 | Medication room, trolley | Medication room, trolley | Bedside | Bedside | Bedside, medication room | Medication room |
| 10 | Medication room; trolley | Trolley | Bedside | Bedside | Bedside, medication room | Medication room |
| 11 | Medication cupboard area | Medication cupboard area, bedside | Bedside | Bedside | Drawer on the unit | Box in desk at nurses’ station |
| 12 | Medication room | Medication room | Medication room | Not stored | Other ward | Pharmacy remove |
| 13 | Medication cupboard area | Medication cupboard area | Medication cupboard area | Patients keep themselves | TTA cupboard | Pharmacy remove |
| 14 | Medication room, trolley | Medication room, trolley | Bedside | Bedside | Medication room | Medication room |
| 15 | Medication room, trolley | Medication room, trolley | Bedside | Bedside | Medication room, bedside | Medication room |
| 16 | Medication room | Medication room | Patient box in medication room | Medication room | Medication room | Medication room |
| 17 | Medication room | Bedside | Bedside | Bedside | None stored | Medication room |
| 18 | Medication room | Bedside | Bedside | Bedside | None stored | Pharmacy remove |
| 19 | Medication room, trolley | Medication room, trolley | Bedside | Bedside | Medication room | Medication room |

| 20 | Medication room, trolley | Trolley | Bedside | Bedside | Medication room | Medication room |
| --- | --- | --- | --- | --- | --- | --- |
| 21 | Medication room, trolley | Medication room, trolley | Bedside | Not stored | Medication room | Medication room |
| 22 | Medication cupboard | Trolley | Bedside | Bedside | Medication cupboard | Clinical room |
| 23 | Medication room | Medication room | Medication room | Patients keep themselves | Not stored | Medication room |
| 24 | Medication room, trolley | Trolley | Trolley | Trolley, patients keep themselves | Medication room | Medication room |
| 25 | Medication room, trolley | Trolley | Trolley | Not stored | Medication room | Medication room |
| 26 | Medication room | Medication room | Medication room | Medication room | Not stored | Medication room |
| 27 | Medication room | Medication room | Medication room | Medication room | Medication room | Medication room |
| 28 | Cupboards by nurses’ station and fluid cupboards | Cupboards by nurses’ station | Cupboards by nurses’ station | Cupboards by nurses’ station | Not stored | Pharmacy remove |

**Table A17. Medication storage practices at hospital site 4**

| **Ward** | **Ward stock** | **Ward stock in use by a specific patient** | **Medication dispensed to patients for inpatient use** | **Patients’ own medication** | **Medication dispensed for discharge** | **Medication for returning to pharmacy** |
| --- | --- | --- | --- | --- | --- | --- |
| 1 | Medication room, trolley | Medication room, trolley | Medication room, trolley | Medication room, trolley | Medication room | Medication room |
| 2 | Medication room, trolley | Medication room | Medication room | Patients keep themselves | Medication room | Medication room |
| 3 | Medication room, trolley | Medication room, trolley | Bedside | Bedside | Medication room | Medication room |
| 4 | Medication room, trolley | Medication room, trolley | Medication room, trolley | Patients keep themselves | Medication room | No particular place |
| 5 | Medication room, cupboard in one bay | Medication room, cupboard in one bay | Medication room | Medication room | Cupboard in bay | Medication room |
| 6 | Medication room, trolley | Trolley | Trolley | Not stored | Not stored | Medication room |
| 7 | Medication room, trolley | Medication room, trolley | Trolley | Not stored | Trolley | Did not know |
| 8 | Medication room, trolley | Trolley | Trolley | Patients keep themselves | Medication room | Medication room |

**Table A18. Drug rounds at hospital site 1**

| **Ward** | **How is medication transported?** | **Where is medication prepared?** |
| --- | --- | --- |
| 1 | Trolley or tray | At the bedside |
| 2 | Already at the bedside | At the bedside |
| 3 | Trolley or tray | At the bedside |
| 4 | Already at the bedside | At the bedside |
| 5 | Tray | At the bedside |
| 6 | Trolley | Medication room and at the bedside |
| 7 | Tray | Medication room and at the bedside |
| 8 | Trolley | Medication room and at the bedside |
| 9 | Trolley | At the bedside |
| 10 | Tray | Medication room and at the bedside |
| 11 | Already at the bedside | At the bedside |
| 12 | Tray | Medication room |
| 13 | Tray | Medication room and at the bedside |
| 14 | By hand in a pot | At the trolley on the unit or at the bedside |
| 15 | Tray | At the bedside |
| 16 | Tray | Medication room and at the bedside |
| 17 | Tray | Medication room and at the bedside |
| 18 | Already at the bedside | At the bedside |
| 19 | Already at the bedside | At the bedside |
| 20 | Tray | At the bedside |
| 21 | Already at the bedside | At the bedside |
| 22 | Already at the bedside | At the bedside |
| 23 | Tray | At the bedside |
| 24 | Tray | Medication room and at the bedside |

**Table A19. Drug rounds at hospital site 2**

| **Ward** | **How is medication transported?** | **Where is medication prepared?** |
| --- | --- | --- |
| 1 | Trolley | At the bedside |
| 2 | Tray | Medication room and at the bedside |
| 3 | Tray | Medication room and at the bedside |
| 4 | Trolley | Medication room and at the bedside |
| 5 | Already at the bedside | At the bedside |
| 6 | Tray | Medication room and at the bedside |
| 7 | Trolley | Medication room and at the bedside |
| 8 | Already at the bedside | At the bedside |
| 9 | Trolley | Medication room and at the bedside |
| 10 | Trolley | At the bedside |
| 11 | Tray | At the bedside |
| 12 | Tray | Medication room |
| 13 | Tray | Medication room and at the bedside |
| 14 | Already at the bedside | At the bedside |
| 15 | Trolley and tray | Medication room and at the bedside |
| 16 | Already at the bedside | At the bedside |
| 17 | Tray | At the bedside |

**Table A20. Drug rounds at hospital site 3**

| **Ward** | **How is medication transported?** | **Where is medication prepared?** |
| --- | --- | --- |
| 1 | Trolley | At the bedside |
| 2 | Trolley | At the bedside |
| 3 | Trolley | At the bedside |
| 4 | Trolley | At the bedside |
| 5 | Trolley | Medication room and at the bedside |
| 6 | Trolley | At the bedside |
| 7 | Trolley | Medication room and at the bedside |
| 8 | Trolley | At the bedside |
| 9 | Tray | Medication room and at the bedside |
| 10 | Trolley | At the bedside |
| 11 | Trolley | In the corridor |
| 12 | Tray | Medication room |
| 13 | Trolley | In the corridor |
| 14 | Trolley or tray | At the bedside |
| 15 | Trolley | At the bedside |
| 16 | Tray | Medication room |
| 17 | Already at the bedside | At the bedside |
| 18 | Already at the bedside | At the bedside |
| 19 | Trolley | At the bedside |
| 20 | Trolley | At the bedside |
| 21 | Tray | At the bedside or in the corridor |
| 22 | Trolley or tray | Medication room and at the bedside |
| 23 | Tray | Medication room |
| 24 | Trolley | At the bedside |
| 25 | Trolley | At the bedside |
| 26 | Tray | At the bedside |
| 27 | Trolley | Medication room and at the bedside |
| 28 | Trolley | At the bedside |

**Table A21. Drug rounds at hospital site 4**

| **Ward** | **How is medication transported?** | **Where is medication prepared?** |
| --- | --- | --- |
| 1 | Trolley | Medication room and at the bedside |
| 2 | Trolley | Medication room and at the bedside |
| 3 | Tray | Medication room and at the bedside |
| 4 | Trolley | Medication room and at the bedside |
| 5 | Tray | Medication room and at the bedside |
| 6 | Trolley | At the bedside |
| 7 | Trolley | At the bedside |
| 8 | Trolley | At the bedside |
